# Action Potential Recording and Pro-arrhythmia Risk Analysis in Human Ventricular Trabeculae

**DOI:** 10.3389/fphys.2017.01109

**Published:** 2018-01-05

**Authors:** Yusheng Qu, Guy Page, Najah Abi-Gerges, Paul E. Miller, Andre Ghetti, Hugo M. Vargas

**Affiliations:** ^1^Integrated Discovery and Safety Pharmacology, Amgen Inc., Thousand Oaks, CA, United States; ^2^AnaBios Corporation, San Diego, CA, United States

**Keywords:** action potential, Pro-arrhythmic risk, human ventricular tissue, TdP assessement, *In-vitro* models

## Abstract

To assess drug-induced pro-arrhythmic risk, especially Torsades de Pointe (TdP), new models have been proposed, such as *in-silico* modeling of ventricular action potential (AP) and stem cell-derived cardiomyocytes (SC-CMs). Previously we evaluated the electrophysiological profile of 15 reference drugs in hESC-CMs and hiPSC-CMs for their effects on intracellular AP and extracellular field potential, respectively. Our findings indicated that SC-CMs exhibited immature phenotype and had the propensity to generate false positives in predicting TdP risk. To expand our knowledge with mature human cardiac tissues for drug-induced pro-arrhythmic risk assessment, human ventricular trabeculae (hVT) from ethically consented organ donors were used to evaluate the effects of the same 15 drugs (8 torsadogenic, 5 non-torsadogenic, and 2 discovery molecules) on AP parameters at 1 and 2 Hz. Each drug was tested blindly with 4 concentrations in duplicate trabeculae from 2 hearts. To identify the pro-arrhythmic risk of each drug, a pro-arrhythmic score was calculated as the weighted sum of percent drug-induced changes compared to baseline in various AP parameters, including AP duration and recognized pro-arrhythmia predictors such as triangulation, beat-to-beat variability and incidence of early-afterdepolarizations, at each concentration. In addition, to understand the translation of this preclinical hVT AP-based model to clinical studies, a ratio that relates each testing concentration to the human therapeutic unbound Cmax (Cmax) was calculated. At a ratio of 10, for the 8 torsadogenic drugs, 7 were correctly identified by the pro-arrhythmic score; 1 was mislabeled. For the 5 non-torsadogenic drugs, 4 were correctly identified as safe; 1 was mislabeled. Calculation of sensitivity, specificity, positive predictive value, and negative predictive value indicated excellent performance. For example, at a ratio of 10, scores for sensitivity, specificity, positive predictive value and negative predictive values were 0.88, 0.8, 0.88 and 0.8, respectively. Thus, the hVT AP-based model combined with the integrated analysis of pro-arrhythmic score can differentiate between torsadogenic and non-torsadogenic drugs, and has a greater predictive performance when compared to human SC-CM models.

## Introduction

The pharmaceutical industry has been conducting studies according to ICH S7B guideline (ICH S7B, 2004) for preclinical assessment and ICH E14 (ICH E14, 2005) guideline for clinical evaluation since 2005 to understand drug-induced pro-arrhythmic risks, especially Torsades de Pointes (TdP). In July of 2013, a novel paradigm, the comprehensive *in-vitro* pro-arrhythmic assessment (CiPA), was proposed (FDA-HESI-Cardiac Safety Research Consortium Workshop, Sager et al., 2014) as a definitive approach for directly assessing pro-arrhythmic risks using *in-vitro* and *in-silico* approaches that incorporate multi-ion channel potencies, i.e., moving beyond hERG potency alone. The CiPA approach for assessing pro-arrhythmic risk is composed of 3 models (Sager et al., 2014), (1) potency determination of 3–7 cardiac ion channels; (2) *in-silico* action potential (AP) modeling; and (3) drug effects on the electrical activity of human stem cell-derived cardiomyocytes (hSC-CM). The CiPA initiative has propelled a multitude of *in-vitro* activities in assay development and validation, in particular in regard to hSC-CM and *in-silico* modeling of ventricular electrophysiology (e.g., Abi-Gerges et al., 2017; Ando et al., 2017; Blinova et al., 2017).

For understanding the potential of hSC-CM in assessing pro-arrhythmic risk, we have tested a set of 15 compounds (ten hERG blockers; four Na channel blockers; one IKs blocker) with a well understood pro-arrhythmic potential in two models of hSC-CM: (1) human embryonic stem cell (hESC)-CM model using traditional patch clamp technique (Qu et al., 2013), and (2) human induced pluripotent stem cell (hiPSC)-CM model using Maestro multi-electrode array (MEA) platform (Qu and Vargas, 2015). This set includes 6 compounds that are included in the CiPA calibration set: dofetilide, sotalol, cisapride, terfanadine, mexiletine, and ranolazine, with 2 in each TdP risk categories (high, intermediate, and low). We found that AP recordings in hESC-CM are sensitive to repolarization delay induced by hERG blockers, but less sensitive for identifying Nav1.5 inhibition, and insensitive to a potent and specific IKs blocker. Consistently, MEA recordings in hiPSC-CM demonstrate that this model would have a high false positive rate when evaluating pro-arrhythmic risk, which could lead to premature termination of drug candidates, a highly undesirable outcome for early safety screening assays. In addition, this model is not able to differentiate Na^+^ channel blockade from hERG blockade due to reduced repolarization reserve in hSC-CM. Our experiences with hSC-CM indicate that pro-arrhythmia risk assessment in hSC-CM is not ready for primetime. Our findings are consistent with the outcome of a recent industry survey conducted by the Safety Pharmacology Society (Authier et al., 2017), which reported that only 21% of responders considered hSC-CM representative of adult cardiomyocytes and provide reliable data as a nonclinical safety assay.

To calibrate the performance of *in-silico* modeling of ventricular AP and hSC-CM, it is important to benchmark adult human ventricular APs, presently regarded as the gold standard for the investigation of pharmacological targets and for the prediction of the pro-arrhythmic potential of novel compounds. A recent publication by Page et al. (2016) described the AP recordings in human ventricular trabeculae for assessing pro-arrhythmia risk by testing 3 TdP agents and 2 non-TdP agents. The TdP risk of these 5 agents were differentiated clearly in this human tissue-based platform by measuring AP-related parameters. To validate this approach independently, we tested and analyzed blindly the same 15 agents that have been tested in hSC-CM (Qu et al., 2013; Qu and Vargas, 2015) in human ventricular trabeculae so that a head-to-head comparison could be performed between electrophysiological recordings in hSC-CM and AP recordings in mature human ventricular tissue.

## Methods

### Donor heart procurement

All human hearts that were used for this study were obtained by legal consent from organ donors in the US. Policies for donor screening and consent are the ones established by the United Network for Organ Sharing. Organizations supplying human tissues to AnaBios follow the standards and procedures established by the US Centers for Disease Control and are inspected biannually by the Department of Health and Human Services. Tissue distribution is governed by internal IRB procedures and compliance with HIPAA regulations regarding patient privacy. All organ donor transfers to AnaBios are fully traceable and periodically reviewed by US Federal authorities.

AnaBios obtained donor hearts from adults aged 17–60 years old. Some donors were trauma victims but the following conditions were excluded: Ejection fraction <45%, HIV, cardiac death, HBV, congenital LQT syndrome, HCV, LOT syndrome, MRSA, downtime >20 min, ongoing infections, positive blood cultures without treatment and 48-h results.

Donor hearts from males and females were harvested using AnaBios' proprietary surgical techniques and tools and were shipped to AnaBios via dedicated couriers. Upon arriving at AnaBios, each heart was assigned a unique identifier number that was reproduced on all relevant medical history files, data entry forms and electronic records.

### Recording of action potentials in human ventricular trabeculae

Tissue dissection: the procedures of tissue dissection and recording were similar to what had been previously described (Page et al., 2016). Briefly, the human heart was transferred into a dissection vessel containing a cold (4°C), fresh proprietary dissection solution. The heart was maintained completely submerged in dissection solution. Ventricular trabeculae were dissected and transferred to the recording chamber.

Recording of APs: The approach used to record APs is similar to that in a recent study (Page et al., 2016). Briefly, a single tissue was mounted into the experimental chamber filled with oxygenated Tyrode's external solution. The temperature of the solution was maintained at 37°C with flow rate at 5 mL per minute. The tissue was allowed to equilibrate for 30–60 min with stimulation (3 V, 3 ms) at a frequency of 1.0 Hz. High impedance borosilicate microelectrodes were prepared with a tip resistance of 10–20 MΩ once filled with 3 M KCl. Upon tissue impalement, the membrane potential was allowed to stabilize (typically, around −85 mV). Tissues with resting membrane potentials more positive than −75 mV were rejected. Bipolar stimulation at 1.5x threshold was applied and recordings were performed in continuous mode with sampling at 20 kHz using ADInstruments and LabChart Software.

Tissue exclusion Criteria: (1). Interruption of perfusion/oxygenation; (2). Absence of Aps following stimulation at baseline; (3). Time frame of drug exposure not respected; (4). Unstable response to stimulation at baseline; (5). Resting membrane potential (RMP) > −75 mV; 7). Maximal amplitude of AP (AMAX) <70 mV; 8). AP duration at 90% repolarization (APD90) <200 ms or >450 ms.

**Figure 1:**
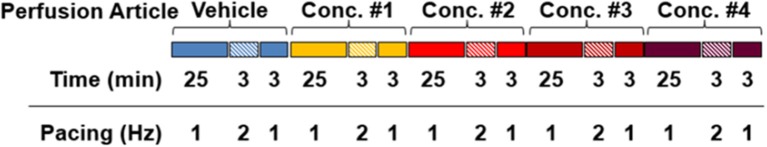
Experimental procedures for each compound tested in each tissue. Baseline APs were collected and assessed for 31 min in vehicle control solution (Tyrode with 0.1% DMSO) at stimulation frequencies of 1 Hz for 25 min, 2 Hz for 3 min and then 1 Hz for 3 min. Following baseline, 4 concentrations of a test compound were applied sequentially and cumulatively. Each concentration was applied for 31 min with the same stimulation paradigm as in control condition.

Experimental Procedure: Each test article was evaluated at 4 concentrations in 4 ventricular trabeculae derived from a minimum of 2 donor hearts. Testing concentrations were chosen based upon human free ETPC, aiming to cover 1–100-fold of human free ETPC. All tissues tested respected the treatment sequence and time course designated in Figure 1. Briefly, following stabilization of each tissue, APs were collected and assessed for 31 min in vehicle control solution (Tyrode with 0.1% DMSO) at stimulation frequencies of 1 Hz for 25 min, 2 Hz for 3 min and then 1 Hz for 3 min. Following this vehicle control period, 4 concentrations of a test compound were applied sequentially and cumulatively. Each concentration was applied for 31 min with the same stimulation sequence as in vehicle controls.

### Data analysis

For each frequency tested, the last 30 APs acquired at the end of the period were averaged for vehicle controls and for each test article concentration. Analysis at 1 Hz included only the last 30 APs of the initial 25-min incubation period. The following AP parameters and pro-arrhythmia variables were analyzed offline upon the completion of recordings:

1. RMP (mV)
2. AMAX (mV)
3. AP duration at percent repolarization (APD20, APD30, APD50, APD60, APD90) (ms)
4. Short term variability analysis of AP duration (STV): Beat-to-beat variability of repolarization was quantified as STV from APD90 Poincare plots over a period of 30 sec. STV for all APDs was calculated as STV = Σ|APDn+1−APDn|/(30×√2), where APD (n) and APD(n+1) are the APDs for the nth AP and the following one, respectively.
5. Triangulation (APD90-APD30)
6. AP instability was calculated for all APD90 values as the standard deviation (SD) of 30 consecutive APs.
7. APD alternans was calculated as the difference for successive Odd and Even APD90 values for 30 consecutive APs.
8. Maximum APD Dispersion was calculated as the difference of Maximum APD90-Minimum APD90 values of 30 consecutive APs.
9. Effective refractory period [ERP(APD)]: Ratio of (APD50-APD20)/APD50 was analyzed to describe ERP of the AP.
10. Beat Escape incidence (%): Electrical stimulus did not trigger an AP following repolarization.
11. Refractoriness Escape incidence (%): Electrical stimulus did not initiate an AP because APD exceeded the inter-stimulus interval.
12. EAD incidence (%): An early afterdepolarization (EAD) was identified as abnormal depolarization during phase 2 or phase 3 of the AP.

Compound effects were quantified relative to the data collected during the vehicle control period (see Figure 1). Threshold values for changes over baseline control for APD30, APD50, APD90, Triangulation and STV at 1 and 2 Hz pacing frequencies have been determined in a previous validation study (Page et al., 2016). Additionally, based on AnaBios historical data, threshold values for changes over baseline control for AP instability, APD alternans, Maximum APD dispersion and ERP (APD) were based upon an effect level of 10%. When applicable, differences were tested for statistical significance using the unpaired Student's *t*-test. A value of *P* < 0.05 was considered significant.

AP parameters and pro-arrhythmia variables were combined into a meaningful, single score to assess the pro-arrhythmic risk of a compound at each concentration tested. The pro-arrhythmic potential of compounds at 1 or 2 Hz was determined by assigning a weighted scale to each variable (Table 1). The maximum score (the sum) calculated at either 1 or 2 Hz was selected as *The Pro-Arrhythmic Potential Score* for each concentration. Based on historical data of this APD assay, a score ≤ 10 indicates a non-pro-arrhythmic potential, while a score ≥ 10 indicates pro-arrhythmic potential.

**Table 1:** Determination of the pro-arrhythmia score.

**Parameter**	**Measurement and calculation**	**Increase (% change) and incidence (%) at 1 Hz**	**Weighted scale**	**Decrease (% change) at 1 Hz**	**Weighted scale**
Action potential duration (APD)	Measured at 20, 30, 50, 70, and 90% repolarization (ADP20, APD30, APD50, APD70, and APD90). APD data were expressed as the mean of 30 consecutive APDs.	0–10 >10–15 >15–20 >20–30 >30–40 >40–50 >50–100 >100	0 1 3 5 7 9 11 13	0–10 >10–15 >15–20 >20–30 >30–40 >40–50 >50–100 >100	0 −1 −3 5 7 9 11 13
Short term variability of APD90 [STV(APD90)]	Beat-to-beat variability of repolarization was quantified as STV from APD Poincaré plots over a period of 30 sec and calculated as STV = Σ|APDn+1−APDn|/(30 × √2), where APD (n) and AP variability D(n + 1) were the APD90s for the nth AP and the following one, respectively.	0–102 >102–120 >120–140 >140–160 >160–180 >180–200 >200	0 2 4 6 8 10 12	0–102 >102-120 >120–140 >140–160 >160–180 >180–200 >200	0 −2 −4 −6 −8 −10 −12
AP triangulation	Calculated as the difference of APD90-APD30.	0–10 >10–15 >15–20 >20–30 >30–40 >40–50 >50–100 >100	0 2 4 6 8 10 12 14	0–10 >10–15 >15–20 >20–30 >30–40 >40–50 >50–100 >100	0 −2 4 6 8 10 12 14
Maximum APD dispersion APD alternans Effective refractory period [ERP(APD)]	Calculated as the difference of Maximum APD90 - Minimum APD90 of 30 consecutive APs. Calculated as the APD difference for successive Odd and Even APD90 of 30 consecutive APs. Ratio of APD50-APD20/APD50	0–10 >10–20 >20–30 >30–40 >40–50 >50–100 >100	0 2 4 6 8 10 12	0–10 >10–20 >20–30 >30–40 >40–50 >50–100 >100	0 −2 −4 −6 −8 −10 −12
APD90 instability	Calculated as the standard deviation (SD) of 30 consecutive APD90	0–10 >10–20 >20–30 >30–40 >40–50 >50–100 >100	0 1 3 5 7 9 11	0–10 >10–20 >20–30 >30–40 >40–50 >50–100 >100	0 −1 −3 −5 −7 −9 −11
AP Escape incidence (%) Refractoriness Escape incidence (%)	Electrical stimulus did not trigger an AP following repolarization. Electrical stimulus did not initiate an AP because APD exceeded the inter-stimulus interval.	0 <10 >10–20% >20–30% >30–40% >40–50% >50–100	0 2 4 6 8 10 12		
EAD incidence (%)	An early afterdepolarization (EAD) was identified as abnormal depolarization during phase 2 or phase 3 of the AP, which was caused by an increase in the frequency of abortive APs before normal repolarization was completed.	0 <10 >10–20 >20–30 >30–40 >40–50 >50–100	0 4 8 12 16 20 24		

*Similar weighted scales were used for STV(APD90) at 2 Hz, although increase or decrease % changes in STV(APD90) were as follows: 0–164%, >164–200%, >200–220%, >220–240%, >240–260%, >260–300% and >200%. A linear weighted scale was assigned to each variable and this assignment took into consideration (i) the role of each variable during pro-arrhythmia and (ii) drug-induced % change or % incidence. To identify the pro-arrhythmic risk of each drug, a pro-arrhythmic score was calculated as the weighted sum of % drug-induced changes in various AP parameters at each concentration. Score was determined at 1 and 2Hz pacing frequencies and the maximum score at 1 or 2Hz was selected as The Representative Score. Based on historical AP data analysis, a score size of 10 was applied to all drugs: score ≤ 10 indicated no pro-arrhythmic potential, while a score >10 indicated a pro-arrhythmic potential. Whereas decrease or increase in weighted scales for each variable indicated anti-pro-arrhythmic potential or potential pro-arrhythmia risk, respectively, a significant decrease in APD90 or triangulation indicated potential pro-arrhythmia risk. Threshold values for changes over baseline control for AP parameters in this human ex-vivo AP model were reported in Page et al. (2016)*.

### Reagents

Sertindole (CAS # 106516-24-9) and Moxifloxacin (151096-09-2) were purchased from ChemPacific (MD, USA), L768673 (Selnick et al., 1997) was purchased from Albany Molecular Research Inc. (N Y, USA), Cisapride (81098-60-4) was from Tocris Bioscience (MO, USA), Ranolazine, Alfuzosin, Mexiletine, Flecainide, Terfenadine, Lamotrigine, DL-sotalol, and Terodiline were from Sigma-Aldrich (MO, USA), Dofetilide was synthesized at American Custom Chemicals Corporation (San Diego, CA), Tolterodine (214601-13-5) was from Toronto Research Chemicals (Toronto, Canada), and AMG 1 was synthesized at Amgen Medicinal Chemistry (Thousand Oaks, CA). All compounds were dissolved in DMSO to make stock solutions.

## Results

### Effect of tolterodine and terodiline on AP in human ventricular trabeculae

Both tolterodine and terodiline are relatively potent hERG blockers (Martin et al., 2006), yet tolterodine is considered safe in the clinic (Malhotra et al., 2007), whereas terodiline was withdrawn from the market due to adverse cardiac events (Thomas et al., 1995). To understand the performance of AP recording and analysis in hVT, Tolterodine and terodiline profiles were characterized side-by-side.

**Figure 2:**
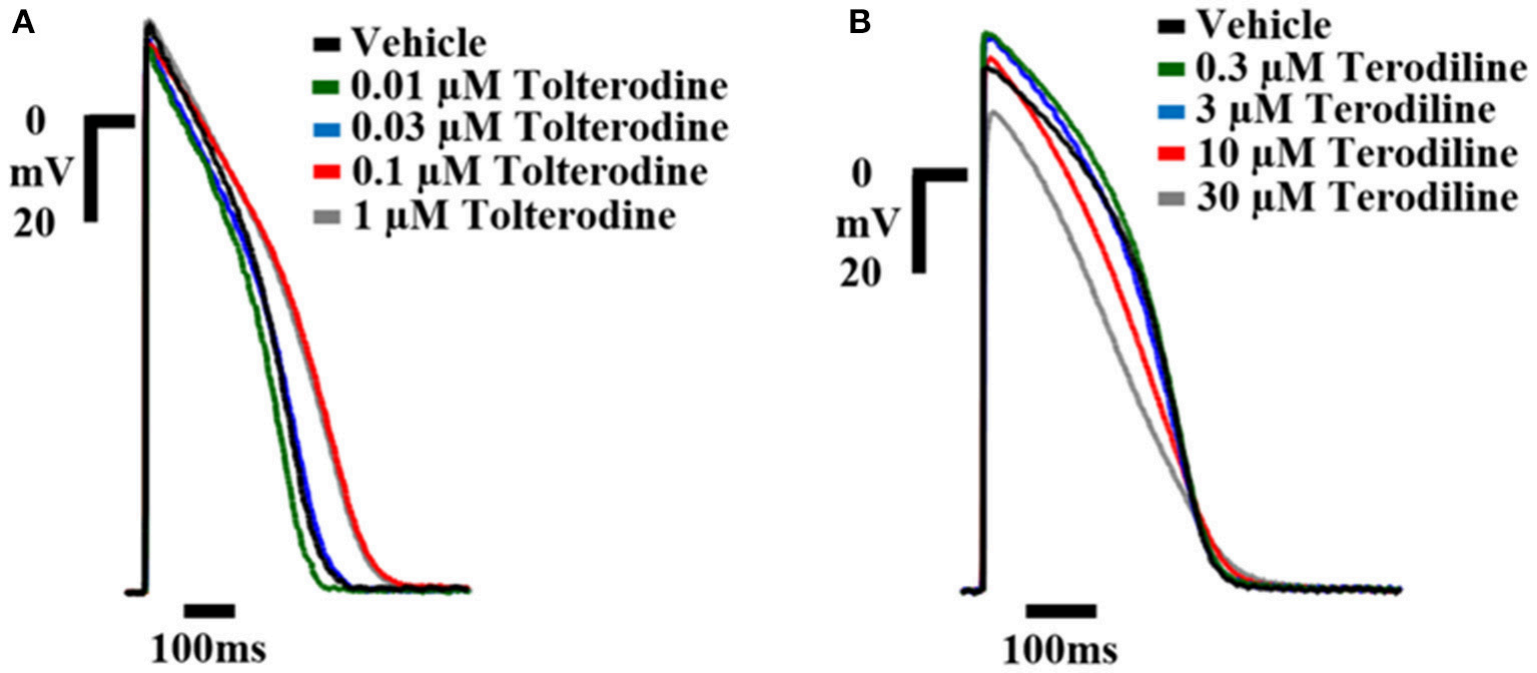
Example AP traces from hVT in control and in the presence of increasing concentrations of tolterodine **(A)** and terodiline **(B)** as labeled. Stimulation was applied at a frequency of 1 Hz. Telterodine was tested at concentrations of 0.01, 0.03, 01, and 1 μM (as shown), and terodiline was tested at concentrations of 0.3, 3, 10, and 30 μM (as shown). Calibration: 20 mV; 100 ms. Tolterodine prolonged phase 3 of AP repolarization AP without prominent effects on AP amplitude, while terodiline decreased AP amplitude, shortened phase 2, lengthened phase 3 of repolarization.

The concentration-dependent effects of tolterodine and terodiline on AP in human ventricular trabeculae are shown in Figure 2, which were recorded separately in 2 example tissues. To understand the translation from *ex-vivo* AP recordings to clinical observations, the testing concentrations were converted to multiple of free effective therapeutic concentrations (ETPC) in the clinic, which equals testing concentrations divided by free ETPC. As shown in Figure 2, tolterodine increased the duration of AP repolarization at 0.1 and 1 μM with predominant prolongations of phase 3 without notably affecting phase 2. In addition, tolterodine had negligible effects on the amplitude of AP up to the highest testing concentration, 1 μM.

On the other hand, terodiline significantly modified the AP morphology by lengthening the phase 3 while shortening the phase 2 of AP repolarization, in addition, terodiline decreased the amplitude of AP, especially at 30 μM.

**Figure 3:**
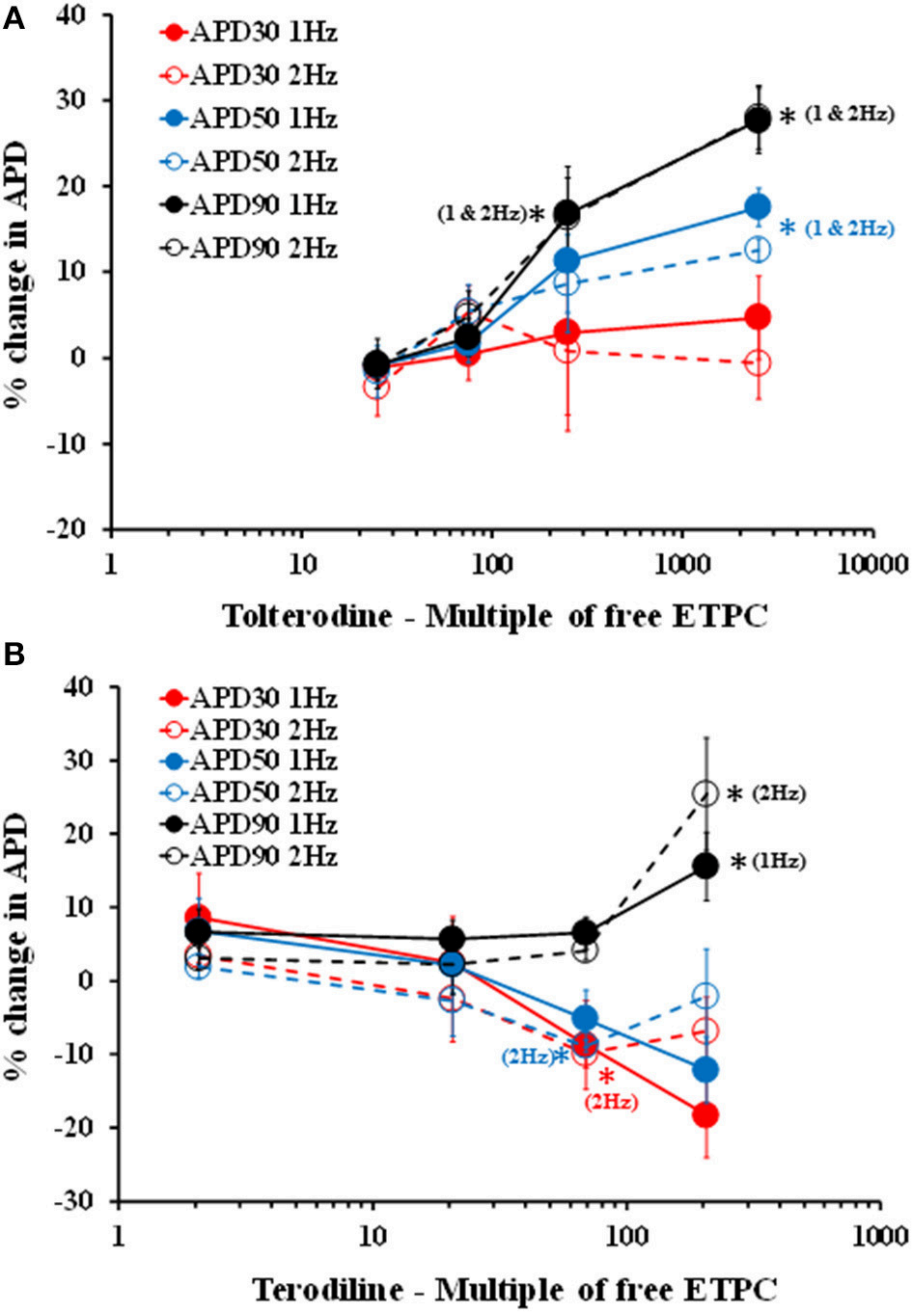
Concentration-dependent changes of APD, including APD30, APD50, and APD90, in the presence of increasing concentrations of tolterodine **(A)** and terodiline **(B)** under stimulation frequencies of 1 and 2 Hz. Tolterodine induced a concentration-dependent increase of APD90 and APD50 with minimal effects on APD30. Terodiline prolonged APD90, but shortened APD50 and APD30. ^*^P < 0.05 versus values from vehicle.

To further analyze the effects of tolterodine and terodiline on hVT AP, percent changes of APD normalized by baseline values, including APD30, APD50, and APD90, were derived at stimulation frequencies of 1 Hz and 2 Hz (Figure 3). Consistent with the observation in Figure 2, tolterodine induced a concentration-dependent increase of APD50 and APD90, which represent the early and late stages of the phase 3 repolarization. On the other hand, APD30, the phase 2 repolarization, was not affected. Effects of terodiline on AP duration exhibited a differing profile compared to tolterodine (Figure 3B). The late stage of phase 3 repolarization, APD90, was prolonged, however, phase 2, APD30, and the early stage of phase 3, APD50, were shortened.

### Effects of tolterodine and terodiline on pro-arrhythmic parameters in human ventricular trabeculae

**Figure 4:**
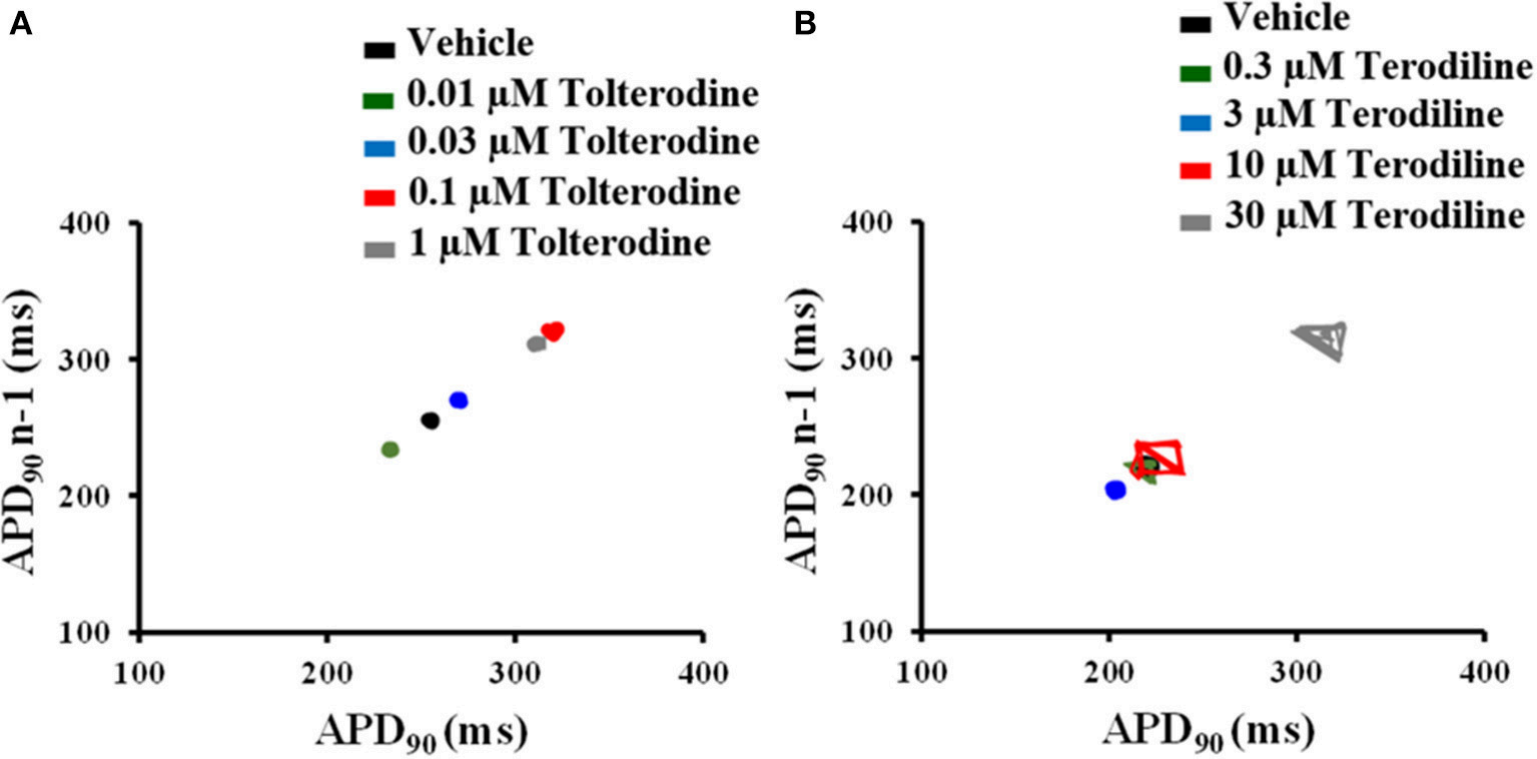
Poincaré plots of APD90 in the presence of increasing concentrations of tolterodine **(A)** and terodiline **(B)** under 2 Hz stimulation. Tolterodine induced minimal APD90 variation, while terodiline produced concentration-dependent increase of APD90 oscillation.

To understand beat-to-beat variability of repolarization, the short-term variability (STV) of AP duration was quantified from APD90 Poincare plots over a period of 30 beats in control and under treatments of tolterodine (A) and terodiline (B) in Figure 4. As shown in Figure 4, under the stimulation frequency of 2 Hz, there is minimal variation of APD90 in the presence of tolterodine, however, significant increase of APD90 oscillation was observed in the presence of terodiline.

**Figure 5:**
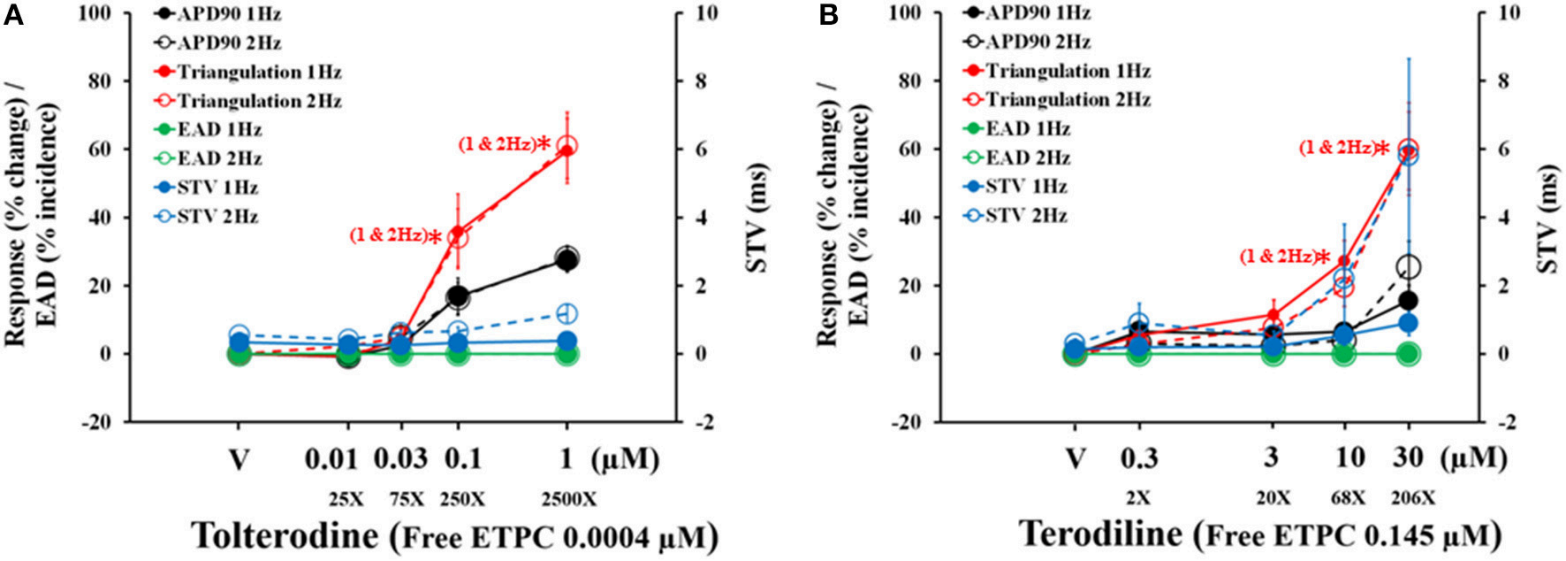
Concentration-dependent plots of mean changes in APD90, triangulation, STV, and EAD incidence in the presence of tolterodine **(A)** and terodiline **(B)** under stimulation frequencies of 1 and 2 Hz. ^*^P < 0.05 versus values from vehicle.

The concentration and frequency-dependent effects of tolterodine and terodiline on pro-arrhythmic parameters, including APD90, triangulation, STV and EAD incidence were described and overlaid in Figure 5. Tolterodine (A) and terodiline (B) did not induce EAD at any testing concentrations under either stimulation frequencies. For both compounds, the changes in APD90 and triangulation are not frequency-dependent, while effects on STV are more prominent under 2 Hz than under 1 Hz, a characteristic of use-dependent effect.

**Figure 6:**
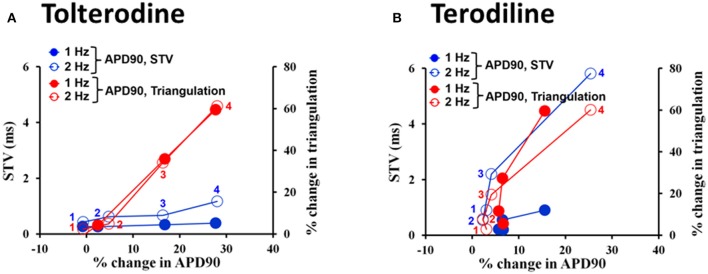
Effects of tolterodine **(A)** and terodiline **(B)** on STV and triangulation as a function of APD90 change under stimulation frequencies of 1 and 2 Hz. Note for tolterodine, there is a linear relationship between triangulation, STV, and APD90. While for terodiline, there is an accelerated change in triangulation compared to changes in APD90 and there is also an accelerated change in STV at a stimulation frequency of 2 Hz.

Effects of tolterodine (A) and terodiline (B) on STV and triangulation as a function of APD90 change were described under stimulation frequencies of 1 Hz and 2 Hz in Figure 6. For both compounds, triangulation became greater with APD90 prolongation, however, the relationship between triangulation and APD90 was linear in the case of tolterodine (Figure 6A). While for terodiline, it is a non-linear relationship with accelerated increase of triangulation with lengthening of APD90. The initial 3 testing concentrations of terodiline, APD90 was not affected by less than 10%, however, change in triangulation increased to 20% and greater. This accelerated increase of triangulation was observed at both stimulation frequencies (Figure 6B). The effect of triangulation was not use-dependent for tolterodine, but reverse use-dependent for terodiline.

Both tolterodine and terodiline produced minimal changes in STV as a function of APD90 prolongation at 1 Hz, and increase of stimulation frequency from 1 to 2 Hz augmented STV (Figure 6). While the increase of STV by tolterodine was minimal as a function of APD90 prolongation, terodiline produced magnified changes of STV at 2 Hz as a function of APD90 prolongation. With less than 10% change in APD90 at the first 3 testing concentrations of terodiline, STV was increased to 2.2 (third concentration) from 0.9 (first conccentration), a 144% increase. Therefore, effects of tolterodine and terodiline on STV were use-dependent, and had distinct profiles as a function of APD90 prolongation.

### Assessment of pro-arrhythmic risk-based on multiples of clinical exposure

**Figure 7:**
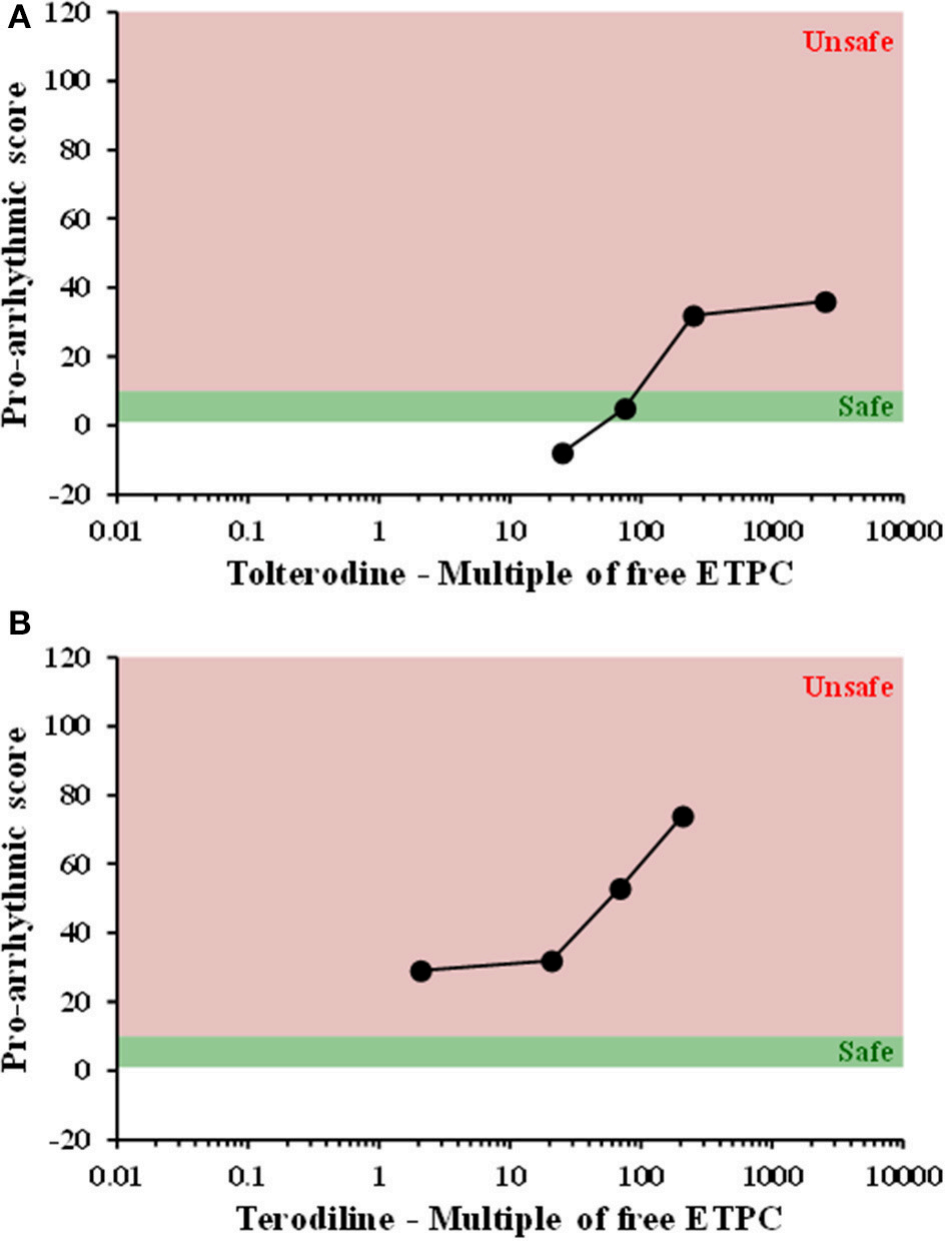
Plots of proarrhythmic score for tolterodine **(A)** and terodiline **(B)** against multiples of free ETPC. Red means unsafe, and green means safe.

**Figure 8:**
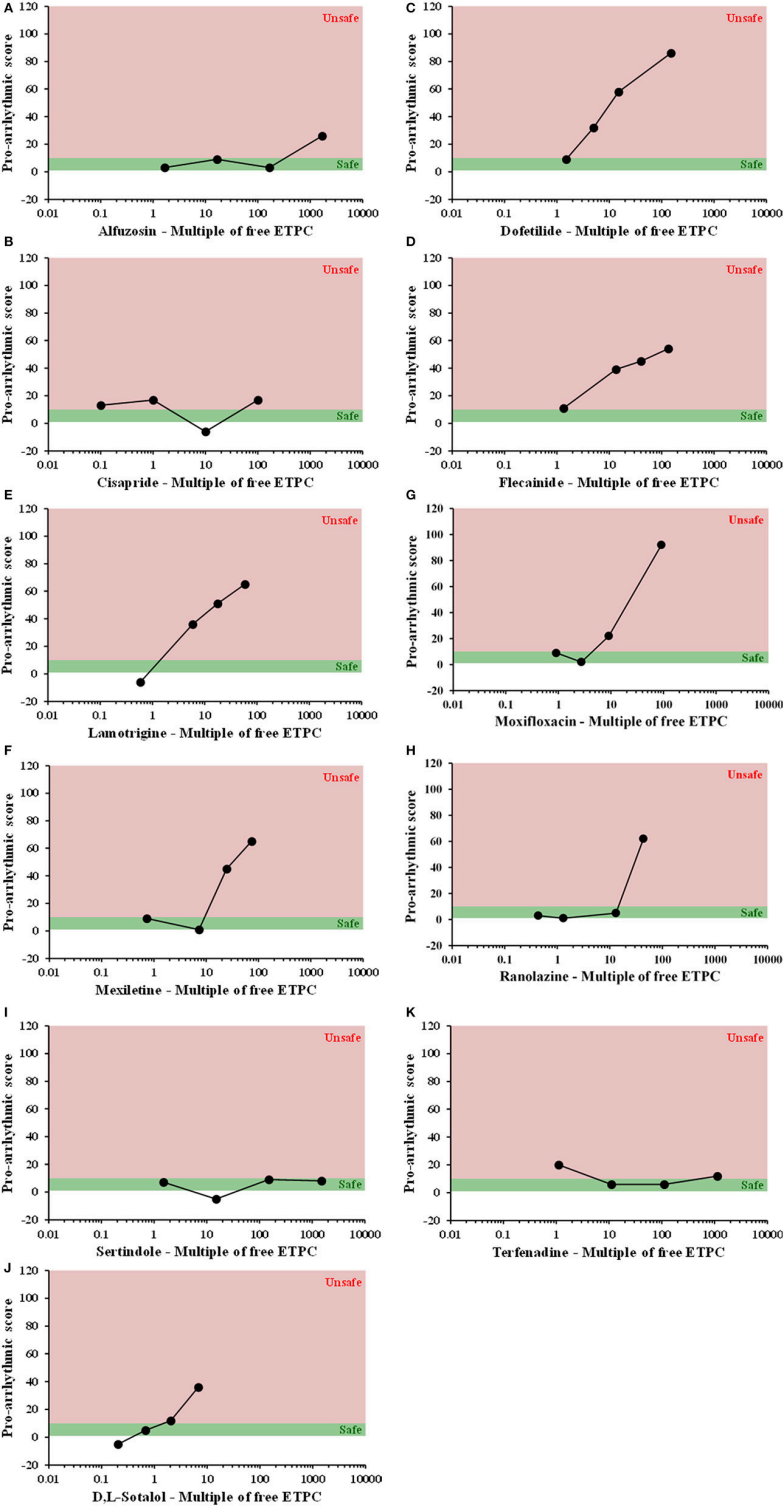
Plots of proarrhythmic score for 11 individual compounds, as labeled, against their free ETPC, **(A)** Alfuzosin, **(B)** Cisapride, **(C)** Dofetilide, **(D)** Flecainide, **(E)** Lamotrigine, **(F)** Mexiletine, **(G)** Moxifloxacin, **(H)** Ranolazine, **(I)** Sertindole, **(J)** Sotalol, and **(K)** Terfenadine. Red means unsafe, and green means safe.

All compounds were evaluated in the same manner as tolterodine and terodiline, the raw data and percent changes of each parameters were described in the Supplementary Material. The value of APD and its derived pro-arrhythmic parameters in human ventricular trabeculae for predicting pro-arrhythmic risk was evaluated by integrating and deriving a pro-arrhythmic score at each testing concentration of each compound (Table 1). Testing concentrations were converted to a multiple of human effective therapeutic plasma concentrations in the unbound fraction. Figure 7 showed the pro-arrhythmic scores as a function of the multiple of free ETPC for tolterodine and terodiline. Figure 8 included the subsequent compounds tested. Two agents, AMG1 and L 768,673 were not displayed due to a lack of human exposure data. Pro-arrhythmic scores were classified into 2 categories based upon the score: unsafe (>10) and safe (<10). Assay performance of pro-arrhythmic scores at a 10-fold of human free ETPC for predicting TdP risk was calculated for the 13 compounds that have human clinical data (Table 2). As shown in Table 2, at a pro-arrhythmic score of > 10, 7 out of 8 compounds are identified correctly as TdP positive, while sertindole was incorrectly identified as a TdP negative. At a pro-arrhythmic score of <10, 4 out of 5 TdP negative compounds were correctly identified, while lamotrigine was identified incorrectly as TdP positive. However, the TdP risk of sertindole and the lack of TdP risk of lamotrigine have been challenged, this will be discussed in detail later.

**Table 2:** Performance of action potential recordings and Pro-arrhythmic score analysis in hVT.

		**Pro-arrythmic score**
	**TdP, Human**	**10X**
Sertindole		
Dofetilide		
Cisapride		
Terfenadine		
Terodiline		
Sotalol (D,L)		
Moxifloxacin		
Flecainide		
Mexiletine		
Tolterodine		
Alfuzosin		
Ranolazine		
Lamotrigine		
Sensitivity		0.88
Specificity		0.8
Positive Predictive Value		0.88
Negative Predictive Value		0.8

Consistent with initial observations, at 10-fold human free ETPC, pro-arrhythmic score had high sensitivity (0.88), specificity (0.8), positive predictive value (0.88), and negative predictive value (0.8) (Table 2).

## Discussion

Drug-induced pro-arrhythmia was assessed by determining the pro-arrhythmic score, a comprehensive score derived from APD and other AP-associated parameters, in hVT. Risk assessment was performed by comparing the pro-arrhythmic score to known clinical exposure, free Cmax. To the best of our knowledge, this is the first blinded study with an extensive reference compound set tested for their effects on ventricular APs in authentic healthy human cardiac tissues. The comprehensive analysis of multiple AP parameters and comparison of the pro-arrhythmic risk of various drugs using a single score in the context of their human clinical exposure clearly distinguished TdP-positive and TdP-negative compounds (Table 2).

As shown in Table 2, at a pro-arrhythmic score of >10, 7 out of 8 compounds were identified correctly as TdP positive, while sertindole was incorrectly identified as a TdP negative. At a pro-arrhythmic score of <10, 4 out of 5 TdP negative compounds were correctly identified, while lamotrigine was identified incorrectly as TdP positive.

### Assay performance depends critically upon categorization of TDP risk in human

Sertindole is an antipsychotic medication developed for the treatment of schizophrenia (Karamatskos et al., 2012). In Europe, sertindole was approved and marketed in 1996, but the drug was withdrawn from the market in 1998 due to concerns of QTc prolongation and potential high risk of fatal arrhythmias in patients. Further clinical epidemiological studies did not provide clear evidence that patients on sertindole were at a significantly increased risk of cardiac arrhythmia and cardiac death (Toumi et al., 2003; Lindström et al., 2005; Spina and Zoccali, 2008). In 2002, sertindole was reintroduced for restricted use in clinical trials. It is a potent hERG blocker with a sub-micromolar IC50 (Rampe et al., 1998; Qu and Vargas, 2015). However, preclinical electrophysiological studies conducted in animal models of pro-arrhythmia have demonstrated that QTc prolongation induced by sertindole does not sufficiently elicit serious and fatal ventricular arrhythmias (Eckardt et al., 2002; Thomsen et al., 2003; Lindström et al., 2005). Therefore, the outcome of current study is consistent with the risk profile of sertindole in both preclinical and clinical settings. If sertindole is categorized as TdP-negative, the assay sensitivity would be perfect with a value of 1.

Lamotrigine has been approved for the treatment of generalized seizures, partial seizures, Lennox–Gastaut syndrome, and bipolar disorder (Rogawski and Loscher, 2004). It inhibits the voltage-gated sodium channels, including Nav1.5, it also inhibits hERG channels (Danielsson et al., 2005). It has an IC50 value of 110 μM for hERG, and 40 μM for Nav1.5 in our previous experiments (Qu and Vargas, 2015). Therefore, at free therapeutic exposure (approximately 17 μM, Dixon et al., 2008) inhibition of hERG channels does not translate into an effect on QT or a pro-arrhythmia risk, which has been demonstrated in a TQT study (Dixon et al., 2008). This correlates well with the current finding that at therapeutic exposure the pro-arrhythmic score is below 10 and lamotrigine is not associated with a pro-arrhythmic risk.

However, when tested at concentrations of 100, 300, and 1,000 μM, lamotrigine blocks hERG channels in addition to inhibition of Nav1.5 and consequently increases its association with pro-arrhythmia risk and conduction delay. The conduction delay further enhances the reverse use-dependence block of hERG channels by lamotrigine. Our findings clearly indicate that this is the case (pro-arrhythmic score is above 40 at these concentrations). There are many clinical case studies for understanding the risk of lamotrigine in terms of its risk of sudden unexpected death, the reported results are conflicting. On one hand, no statistically significant difference in rate of sudden unexpected death between lamotrigine and control groups (e.g., Tomson et al., 2013) has been reported. On the other hand, Aurlien et al. (2012) have shown the evidence that incidence of sudden unexpected death was significantly higher among female patients with epilepsy who were being treated with lamotrigine than among female patients with epilepsy who were not taking lamotrigine. In addition, FDA requires warning labels on the risk of sudden unexpected death in association with the use of lamotrigine. Clinically, overdose of lamotrigine has been shown to be linked to QTc prolongation and pro-arrhythmic risk (e.g., Chavez et al., 2015). In summary, pro-arrhythmia classification of lamotrigine depends on its exposure level and safety margin. At super-therapeutic concentrations, lamotrigine is associated with arrhythmic risk. If lamotrigine is classified as pro-arrhythmic, then the outcome of our current study is perfect with a specificity of 1.

### Pro-arrhythmia risk assessment in mature human cardiac tissue with sharp electrode AP recording: superior to stem-cell derived cardiomyocytes with MEA recording

Previously, we have tested the same 15 compounds and characterized their pharmacological profiles in human induced pluripotent stem cell derived cardiomyocytes (hiPSC-CM) with extracellular field potential recordings (Qu and Vargas, 2015). Our study results indicate that hiPSC-CM would have a high false positive rate when evaluating pro-arrhythmic risk (Table 3). In contrast, current study using the same 15 compounds demonstrated excellent assay performance with sensitivity, specificity, positive predictive value, and negative predictive value all 0.8 and above (Tables 2, 3). This side-by-side comparison with the same set of compounds gives us a greater understanding of the models tested. The results provide us with more confidence that authentic mature human cardiac tissue with sharp electrode AP recording is superior to stem-cell derived cardiomyocytes with MEA recording in assessing a drug's pro-arrhythmic risk.

**Table 3:** Comparison of Assay Performance.

	**TdP, Human**	**iPSC-CM**	**iPSC-CM**	**hVT**
		**Field Potential duration**	**Early After depolarization**	**Arrythmic score**
		**10X**	**10X**	**10X**
Sertindole				
Dofetilide				
Cisapride				
Terfenadine				
Terodiline				
Sotalol (D,L)				
Moxifloxacin				
Flecainide				
Mexiletine				
Tolterodine				
Alfuzosin				
Ranolazine				
Lamotrigine				
Sensitivity		0.88	0.38	0.88
Specificity		0.6	1	0.8
Positive predictive value		0.78	1	0.88
Negative predictive value		0.75	0.5	0.8

### EAD is a rare electrical event in normal human ventricular trabeculae

Early afterdepolarization of ventricular myocytes represents a trigger event that has been implicated as the primary mechanism for ventricular arrhythmia induction in acquired and congenital long QT syndromes, including TdP (Weiss et al., 2010). Interestingly, at the concentration ranges used in our study, some of them are at greater than 100-fold of human free therapeutic Cmax, EAD was only detected in one trabecula, which was under the treatment of moxifloxacin at 91-fold hETPC. None of the other TdP-positive compounds elicited EAD in hVT. Two compounds, dofetilide and sotalol, have previously been tested in a similar experimental design (Page et al., 2016). Lack of EAD was also observed for sotalol. For dofetilide, Page et al. (2016) had observed EAD in 9 out of 21 trabeculae at a testing concentration of 0.1 uM, a 33% incidence. In the current study, dofetilide was tested at 0.003, 0.01, 0.03, and 0.3 μM without any EAD in a total of 4 trabeculae. However, dofetilide did prolong APD starting at 0.003 uM in a concentration-dependent manner with 105% increase of APD90 at 0.3 uM (data not shown), which is comparable with the magnitude of APD90 prolongation observed at 0.1 uM in Page et al. (2016) (~100%). The lack of EAD in the current study could be due to the combination of low incidence (33%) and much smaller number of trabeculae (n = 4) compared to the previous study (Page et al., 2016). Therefore, EAD incidence in AP recordings is not a sensitive biomarker for pro-arrhythmic risk in normal human ventricular trabeculae, false negative rate would be very high if pro-arrhythmic risk was based solely upon EAD incidence.

### An integrated analysis of AP and associated parameters is powerful in differentiating TDP-positive from TDP-negative agents

In analyzing electrophysiological data, multiple endpoints can be derived. For pro-arrhythmic risk assessment, it's a major challenge to determine which endpoint is more important and has more predictive value. Combinations of various endpoints in a weighted manner with expression as an integrated arrhythmic score have been previously successfully used in the A-V ablated isolated rabbit heart model (Hondeghem and Hoffman, 2003; Hondeghem et al., 2003; Lawrence et al., 2006). A similar approach has been taken for the current study with a quantitative arrhythmic score determined by combining all parameters of AP and AP-related parameters, including APD30, APD50, APD90, triangulation, STV, AP instability, APD alternans, Maximum APD dispersion and ERP, etc (details shown in Table 1). In addition, pro-arrhythmic scores are considered in relation to human free ETPC and at multiples above the highest free ETPC. The validation with 8 TdP-positive and 5 TdP-negative agents has demonstrated that this approach is able to differentiate the positive from the negative drugs with high sensitivity and specificity values (Tables 2, 3).

Take an example of tolterodine and terodiline. Both agents are relatively potent hERG blockers (Martin et al., 2006), yet tolterodine is considered safe in the clinic (Malhotra et al., 2007), whereas terodiline was withdrawn from the market because of adverse cardiac events (Thomas et al., 1995). We tested both compounds in ion channel assays, including hERG, Nav1.5, and L-type Ca channel assays (Qu and Vargas, 2015), confirming that both agents are potent hERG channel blockers. In human ventricular trabeculae, both compounds increased AP duration, increased triangulation and short-term variability (Figures 2–5). If examining each of the parameter in isolation, it's difficult to distinguish one from the other in regard to their pro-arrhythmic potentials. When a single pro-arrhythmic score was derived by combining all the parameters in a weighted manner and then related to their human free ETPC, their TdP risks were clearly separated (Figures 7A,B). This case study indicates that AP recordings in hVT combined with analysis of pro-arrhythmic score can differentiate agents that inhibit hERG with significant QTc prolongation and associate with TdP risk, such as terodiline, from agents that inhibit hERG with significant QTc prolongation but not associated with TdP risk, such as tolterodine.

## Limitations

There were several limitations in this study: (1) The tissue in this study came from the trabeculae of the left and right ventricles, which may or may not represent the characteristics of the entire ventricles, because myocardium from different regions of the heart reflect the specialized electrophysiological functions of the region, have different configurations of APs (Schram et al., 2002); To our knowledge, ion channel (hERG, SCN5A, KvLQT1, and KCNE1) distribution at the gene and protein levels in human ventricular trabeculae has not been published. However, it has been shown that SCN5A expression is greater in the endo-than the epi- myocardium, which cause the maximal rate of depolarization (dV/dtmax) higher in the endo-myocardium than in the epi-myocardium (Gaborit et al., 2007). Ikr (hERG) is expressed throughout the myocardium of the human heart with higher expression in epimyocardium than in endomyocardium (Szabo et al., 2005). A more relevant study was performed in human ventricular trabeculae (Jost et al., 2005) that recorded Ikr and Iks (KvLQT1 + KCNE1) with patch clamp technique and specific blockers of Ikr and Iks were used for inhibiting the currents. In addition, this study and a recent study (Jost et al., 2013) showed that there is a robust prolongation of APs in human ventricular trabeculae in response to specific hERG blockers, therefore it is reasonable to postulate that there is abundant hERG in human ventricular trabeculae. (2) Our evaluation was limited by the trabeculae sample size utilized for each concentration. In the previous study (Page et al., 2016), it was recommended that a sample size of at least 2 hearts and 3 trabeculae per heart is necessary to detect drug-related AP changes. In the current study, 2 hearts and 2 trabeculae from each heart were tested for each concentration, a design may not be sufficient for small drug-induced changes; (3) Our conclusion are limited by the number of agents tested, a larger panel of test compounds, including both TdP-positive and TdP-negative standard compounds, would provide increased confidence, and assist in understanding the performance of AP recordings in hVT and analysis of pro-arrhythmic score as a new model for assessing pro-arrhythmic risk; (4) A single calculation of human free ETPC data is a limitation, because there are multiple sources for clinical exposure data, which could introduce selection bias into the ratio calculation.

## Conclusions

This study has tested multiple compounds in authentic human ventricular tissue for their effects on AP, and subsequent determination of pro-arrhythmic scores by calculating the weighted sum of drug-induced changes in various AP parameters and pro-arrhythmia variables. The outcome reported here has demonstrated that this approach yields better performance compared to hSC-CM. Importantly, this approach is able to differentiate agents that inhibit hERG with significant QTc prolongation and associate with TdP risk from agents that inhibit hERG with significant QTc prolongation but not associated with TdP risk.

Therefore, use of primary human cardiac tissues to evaluate pro-arrhythmia risk *in vitro* (Page et al., 2016) could prevent the confounding influences of the embryonic ion channel expression and spontaneous beat rate observed with hSC-CM, and enable a robust and definitive electrophysiological evaluation in mature ventricular myocytes. The performance characteristics of mature ventricular tissue shown here surpass the reliability of iPSC-CM for pro-arrhythmia detection, which give us confidence in employing them for cardiac safety assessment. While the use of mature cardiac tissues does not provide a good screening tool due to the low throughput nature of the assay and the requirement for large numbers of human hearts, this limitation may be overcome by placing this model later in the drug development process, i.e., used only for secondary or Supplemental Purposes.

## Author contributions

YQ: Designed the experiment, wrote the manuscript. HV: designed the experiment, wrote and reviewed the manuscript. GP: performed the study and analyzed data. NA-G: performed the study, analyzed data, wrote and reviewed the manuscript. PM and AG: wrote and reviewed the manuscript.

### Conflict of interest statement

YQ and HV were employed by company Amgen Inc. GP, NA-G, PM, and AG were employed by company AnaBios Corporation. All authors declare no competing interests.
