# Supplementary material for: Action Potential Recording and Pro-arrhythmia Risk Analysis in Human Ventricular Trabeculae
Source: Front Physiol. 2018 Jan 5;8:1109. doi: 10.3389/fphys.2017.01109 (PMC5760531; doi:10.3389/fphys.2017.01109)
Supplement: Supplementary file 1 [file DataSheet1.DOCX]

**Supplementary Material**

Action Potential Recording and Pro-arrhythmia Risk Analysis in Human Ventricular Trabeculae

Yusheng Qu^1^, Guy Page^2^, Najah Abi-Gerges^2^, Paul E Miller^2^, Andre Ghetti^2^ and Hugo M. Vargas^1^

^1^Integrated Discovery and Safety Pharmacology, Amgen Inc., Thousand Oaks, CA 91320, USA; ^2^AnaBios Corporation, San Diego, CA 92109, USA

# RESULTS DOFETILIDE

Averaged raw data and averaged percent change data for vehicle control and for dofetilide at 1 and 2 Hz are summarized for each concentration tested in the tables. Data represent the mean of n=4 trabeculae tested in the experimental series.

## **1.1 SUMMARY DOFETILIDE AT 1Hz**

### 1.1.1: AVERAGE RAW DATA OF ACTION POTENTIAL PARAMETERS FOR VEHICLE CONTROL AND DOFETILIDE AT 1Hz ON VENTRICULAR TRABECULAE AS A FUNCTION OF THE MULTIPLE OF FREE ETPC (0.002µM)

| **Parameters** | **Control** | **1.5-FOLD** | **5-FOLD** | **15-FOLD** | **150-FOLD** |
| --- | --- | --- | --- | --- | --- |
| **APD_30_** | 137 | 146 | 152 | 170 | 190 |
| **APD_50_** | 179 | 195 | 211 | 252 | 311 |
| **APD_90_** | 249 | 274 | 303 | 385 | 510 |
| **AMAX** | 102 | 106 | 107 | 106 | 107 |
| **RMP** | -91 | -91 | -91 | -91 | -89 |
| **Triangulation** | 111 | 128 | 151 | 215 | 320 |
| **STV** | 0.44 | 0.57 | 0.47 | 0.56 | 1.06 |
| **Alternans** | 0.67 | 0.84 | 0.63 | 0.85 | 1.71 |
| **ERP** | 0.41 | 0.43 | 0.47 | 0.52 | 0.59 |
| **Max Dispersion** | 2.62 | 3.05 | 3.93 | 3.58 | 6.48 |
| **SD** | 28 | 25 | 48 | 61 | 87 |
| **Beat Escape** | 0 | 0 | 0 | 0 | 0 |
| **Refractoriness Escape** | 0 | 0 | 0 | 0 | 0 |
| **EADs** | 0 | 0 | 0 | 0 | 0 |

### 1.1.2: AVERAGE PERCENTAGE CHANGES COMPARED TO VEHICLE CONTROL FOR ACTION POTENTIAL PARAMETERS FOR DOFETILIDE AT 1Hz ON VENTRICULAR TRABECULAE AS A FUNCTION OF THE MULTIPLE OF FREE ETPC (0.002µM)

###

| **Parameters** | **1.5-FOLD** | **5-FOLD** | **15-FOLD** | **150-FOLD** |
| --- | --- | --- | --- | --- |
| **APD_30_** | 8 | 12 | 26 | 40 |
| **APD_50_** | 10 | 19 | 42 | 74 |
| **APD_90_** | 10 | 22 | 55 | 105 |
| **AMAX** | 5 | 6 | 4 | 6 |
| **RMP** | 1 | 1 | 1 | -2 |
| **Triangulation** | 14 | 34 | 91 | 186 |
| **STV** | 33 | 4 | 48 | 279 |
| **Alternans** | 26 | -5 | 27 | 155 |
| **ERP** | 4 | 13 | 26 | 42 |
| **Max Dispersion** | 16 | 50 | 36 | 147 |
| **SD** | -9 | 71 | 116 | 212 |
| **Beat Escape** | 0 | 0 | 0 | 0 |
| **Refractoriness Escape** | 0 | 0 | 0 | 0 |
| **EADs** | 0 | 0 | 0 | 0 |

## **1.2 SUMMARY DOFETILIDE AT 2Hz**

### 1.2.1: AVERAGE RAW DATA OF ACTION POTENTIAL PARAMETERS FOR VEHICLE CONTROL AND DOFETILIDE AT 2Hz ON VENTRICULAR TRABECULAE AS A FUNCTION OF THE MULTIPLE OF FREE ETPC (0.002µM)

| **Parameters** | **Control** | **1.5-FOLD** | **5-FOLD** | **15-FOLD** | **150-FOLD** |
| --- | --- | --- | --- | --- | --- |
| **APD_30_** | 101 | 104 | 107 | 115 | 140 |
| **APD_50_** | 135 | 142 | 155 | 172 | 227 |
| **APD_90_** | 200 | 216 | 251 | 296 | 411 |
| **AMAX** | 98 | 102 | 100 | 101 | 101 |
| **RMP** | -90 | -90 | -88 | -88 | -86 |
| **Triangulation** | 98 | 112 | 145 | 181 | 271 |
| **STV** | 0.74 | 0.75 | 0.90 | 1.56 | 33.27 |
| **Alternans** | 1.05 | 1.04 | 1.31 | 2.04 | 46.85 |
| **ERP** | 0.42 | 0.44 | 0.49 | 0.52 | 0.56 |
| **Max Dispersion** | 4.12 | 3.88 | 4.33 | 8.88 | 55.30 |
| **SD** | 15 | 14 | 45 | 39 | 134 |
| **Beat Escape** | 0 | 0 | 0 | 0 | 1 |
| **Refractoriness Escape** | 0 | 0 | 0 | 0 | 0 |
| **EADs** | 0 | 0 | 0 | 0 | 0 |

### 1.2.2: AVERAGE PERCENTAGE CHANGES COMPARED TO VEHICLE CONTROL FOR ACTION POTENTIAL PARAMETERS FOR DOFETILIDE AT 2Hz ON VENTRICULAR TRABECULAE AS A FUNCTION OF THE MULTIPLE OF FREE ETPC (0.002µM)

| **Parameters** | **1.5-FOLD** | **5-FOLD** | **15-FOLD** | **150-FOLD** |
| --- | --- | --- | --- | --- |
| **APD_30_** | 4 | 5 | 13 | 36 |
| **APD_50_** | 6 | 15 | 28 | 65 |
| **APD_90_** | 8 | 25 | 48 | 103 |
| **AMAX** | 5 | 3 | 4 | 4 |
| **RMP** | 0 | -1 | -2 | -4 |
| **Triangulation** | 14 | 46 | 84 | 175 |
| **STV** | -2 | 37 | 133 | 2231 |
| **Alternans** | 0 | 25 | 95 | 4383 |
| **ERP** | 5 | 17 | 24 | 34 |
| **Max Dispersion** | -6 | 5 | 115 | 1241 |
| **SD** | -5 | 197 | 158 | 788 |
| **Beat Escape** | 0 | 0 | 0 | 25 |
| **Refractoriness Escape** | 0 | 0 | 0 | 0 |
| **EADs** | 0 | 0 | 0 | 0 |

# 2. RESULTS TOLTERODINE

Averaged raw data and averaged percent change data for vehicle control and for tolterodine at 1 and 2 Hz are summarized for each concentration tested in the tables. Data represent the mean of n=4 trabeculae tested in the experimental series.

## **2.1 SUMMARY TOLTERODINE AT 1Hz**

### 2.1.1: AVERAGE RAW DATA OF ACTION POTENTIAL PARAMETERS FOR VEHICLE CONTROL AND TOLTERODINE AT 1Hz ON VENTRICULAR TRABECULAE AS A FUNCTION OF THE MULTIPLE OF FREE ETPC (0.0004µM)

| **Parameters** | **Control** | **25-FOLD** | **75-FOLD** | **250-FOLD** | **2500-FOLD** |
| --- | --- | --- | --- | --- | --- |
| **APD_30_** | 188 | 186 | 190 | 192 | 195 |
| **APD_50_** | 251 | 248 | 256 | 279 | 294 |
| **APD_90_** | 323 | 320 | 331 | 378 | 411 |
| **AMAX** | 110 | 110 | 113 | 107 | 112 |
| **RMP** | -87 | -90 | -93 | -90 | -91 |
| **Triangulation** | 135 | 134 | 141 | 186 | 215 |
| **STV** | 0.37 | 0.32 | 0.28 | 0.38 | 0.40 |
| **Alternans** | 0.52 | 0.39 | 0.41 | 0.53 | 0.63 |
| **ERP** | 0.46 | 0.46 | 0.47 | 0.53 | 0.55 |
| **Max Dispersion** | 3.20 | 2.33 | 1.97 | 2.80 | 2.90 |
| **SD** | 31 | 30 | 43 | 58 | 26 |
| **Beat Escape** | 0 | 0 | 0 | 0 | 0 |
| **Refractoriness Escape** | 0 | 0 | 0 | 0 | 0 |
| **EADs** | 0 | 0 | 0 | 0 | 0 |

### 2.1.2: AVERAGE PERCENTAGE CHANGES COMPARED TO VEHICLE CONTROL FOR ACTION POTENTIAL PARAMETERS FOR TOLTERODINE AT 1Hz ON VENTRICULAR TRABECULAE AS A FUNCTION OF THE MULTIPLE OF FREE ETPC (0.0004µM)

| **Parameters** | **25-FOLD** | **75-FOLD** | **250-FOLD** | **2500-FOLD** |
| --- | --- | --- | --- | --- |
| **APD_30_** | -1 | 0 | 3 | 5 |
| **APD_50_** | -1 | 2 | 11 | 18 |
| **APD_90_** | -1 | 2 | 17 | 28 |
| **AMAX** | 0 | 3 | -2 | 2 |
| **RMP** | 4 | 8 | 4 | 5 |
| **Triangulation** | -1 | 4 | 36 | 59 |
| **STV** | -14 | -16 | 12 | 41 |
| **Alternans** | -27 | -23 | 1 | 21 |
| **ERP** | 0 | 2 | 15 | 19 |
| **Max Dispersion** | -27 | -38 | -12 | -9 |
| **SD** | 0 | 39 | 90 | -16 |
| **Beat Escape** | 0 | 0 | 0 | 0 |
| **Refractoriness Escape** | 0 | 0 | 0 | 0 |
| **EADs** | 0 | 0 | 0 | 0 |

## **2.2 SUMMARY TOLTERODINE AT 2Hz**

### 2.2.1: AVERAGE RAW DATA OF ACTION POTENTIAL PARAMETERS FOR VEHICLE CONTROL AND TOLTERODINE AT 2Hz ON VENTRICULAR TRABECULAE AS A FUNCTION OF THE MULTIPLE OF FREE ETPC (0.0004µM)

| **Parameters** | **Control** | **25-FOLD** | **75-FOLD** | **250-FOLD** | **2500-FOLD** |
| --- | --- | --- | --- | --- | --- |
| **APD_30_** | 135 | 131 | 143 | 136 | 134 |
| **APD_50_** | 187 | 184 | 197 | 203 | 210 |
| **APD_90_** | 256 | 254 | 268 | 299 | 327 |
| **AMAX** | 108 | 107 | 113 | 106 | 107 |
| **RMP** | -85 | -91 | -95 | -90 | -90 |
| **Triangulation** | 121 | 123 | 126 | 163 | 193 |
| **STV** | 0.59 | 0.48 | 0.61 | 0.72 | 1.03 |
| **Alternans** | 0.84 | 0.66 | 0.87 | 0.98 | 1.38 |
| **ERP** | 0.48 | 0.49 | 0.48 | 0.54 | 0.56 |
| **Max Dispersion** | 3.00 | 2.38 | 2.93 | 4.08 | 4.05 |
| **SD** | 20 | 21 | 27 | 42 | 22 |
| **Beat Escape** | 0 | 0 | 0 | 0 | 0 |
| **Refractoriness Escape** | 0 | 0 | 0 | 0 | 0 |
| **EADs** | 0 | 0 | 0 | 0 | 0 |

### 2.2.2: AVERAGE PERCENTAGE CHANGES COMPARED TO VEHICLE CONTROL FOR ACTION POTENTIAL PARAMETERS FOR TOLTERODINE AT 2Hz ON VENTRICULAR TRABECULAE AS A FUNCTION OF THE MULTIPLE OF FREE ETPC (0.0004µM)

###

| **Parameters** | **25-FOLD** | **75-FOLD** | **250-FOLD** | **2500-FOLD** |
| --- | --- | --- | --- | --- |
| **APD_30_** | -3 | 5 | 1 | -1 |
| **APD_50_** | -2 | 5 | 9 | 13 |
| **APD_90_** | -1 | 5 | 16 | 28 |
| **AMAX** | 0 | 5 | -1 | 0 |
| **RMP** | 7 | 11 | 5 | 5 |
| **Triangulation** | 2 | 5 | 34 | 61 |
| **STV** | -20 | 10 | 25 | 94 |
| **Alternans** | -21 | 4 | 17 | 64 |
| **ERP** | 2 | -1 | 12 | 17 |
| **Max Dispersion** | -21 | -2 | 36 | 35 |
| **SD** | 8 | 36 | 113 | 12 |
| **Beat Escape** | 0 | 0 | 0 | 0 |
| **Refractoriness Escape** | 0 | 0 | 0 | 0 |
| **EADs** | 0 | 0 | 0 | 0 |

# 3. RESULTS TERODILINE

Averaged raw data and averaged percent change data for vehicle control and for terodiline at 1 and 2 Hz are summarized for each concentration tested in the tables. Data represent the mean of n=4 trabeculae tested in the experimental series.

## **3.1 SUMMARY TERODILINE AT 1Hz**

### 3.1.1: AVERAGE RAW DATA OF ACTION POTENTIAL PARAMETERS FOR VEHICLE CONTROL AND TERODILINE AT 1Hz ON VENTRICULAR TRABECULAE AS A FUNCTION OF THE MULTIPLE OF FREE ETPC (0.145µM)

| **Parameters** | **Control** | **2-FOLD** | **21-FOLD** | **69-OLD** | **207-FOLD** |
| --- | --- | --- | --- | --- | --- |
| **APD_30_** | 141 | 151 | 142 | 126 | 113 |
| **APD_50_** | 190 | 200 | 192 | 178 | 164 |
| **APD_90_** | 255 | 270 | 268 | 270 | 293 |
| **AMAX** | 108 | 111 | 110 | 108 | 102 |
| **RMP** | -92 | -93 | -94 | -96 | -96 |
| **Triangulation** | 113 | 119 | 126 | 144 | 180 |
| **STV** | 0.14 | 0.20 | 0.21 | 0.55 | 0.90 |
| **Alternans** | 0.20 | 0.25 | 0.29 | 0.71 | 1.31 |
| **ERP** | 0.46 | 0.44 | 0.45 | 0.48 | 0.49 |
| **Max Dispersion** | 1.38 | 1.68 | 1.58 | 3.35 | 5.55 |
| **SD** | 42 | 31.3 | 34.6 | 36.6 | 43.9 |
| **Beat Escape** | 0 | 1 | 0 | 1 | 1 |
| **Refractoriness Escape** | 0 | 0 | 0 | 0 | 0 |
| **EADs** | 0 | 0 | 0 | 0 | 0 |

### 3.1.2: AVERAGE PERCENTAGE CHANGES COMPARED TO VEHICLE CONTROL FOR ACTION POTENTIAL PARAMETERS FOR TERODILINE AT 1Hz ON VENTRICULAR TRABECULAE AS A FUNCTION OF THE MULTIPLE OF FREE ETPC (0.145µM)

###

| **Parameters** | **2-FOLD** | **21-FOLD** | **69-OLD** | **207-FOLD** |
| --- | --- | --- | --- | --- |
| **APD_30_** | 9 | 2 | -9 | -18 |
| **APD_50_** | 7 | 2 | -5 | -12 |
| **APD_90_** | 7 | 6 | 7 | 16 |
| **AMAX** | 2 | 2 | 0 | -5 |
| **RMP** | 2 | 2 | 4 | 5 |
| **Triangulation** | 5 | 12 | 27 | 60 |
| **STV** | 119 | 58 | 254 | 545 |
| **Alternans** | 27 | 45 | 257 | 554 |
| **ERP** | -4 | -2 | 5 | 6 |
| **Max Dispersion** | 22 | 15 | 144 | 304 |
| **SD** | -26 | -18 | -13 | 4 |
| **Beat Escape** | 25 | 0 | 25 | 25 |
| **Refractoriness Escape** | 0 | 0 | 0 | 0 |
| **EADs** | 0 | 0 | 0 | 0 |

## **3.2 SUMMARY TERODILINE AT 2Hz**

### 3.2.1: AVERAGE RAW DATA OF ACTION POTENTIAL PARAMETERS FOR VEHICLE CONTROL AND TERODILINE AT 2Hz ON VENTRICULAR TRABECULAE AS A FUNCTION OF THE MULTIPLE OF FREE ETPC (0.145µM)

| **Parameters** | **Control** | **2-FOLD** | **21-FOLD** | **69-OLD** | **207-FOLD** |
| --- | --- | --- | --- | --- | --- |
| **APD_30_** | 112 | 115 | 108 | 100 | 104 |
| **APD_50_** | 154 | 157 | 150 | 140 | 151 |
| **APD_90_** | 219 | 225 | 223 | 228 | 276 |
| **AMAX** | 108 | 109 | 108 | 106 | 100 |
| **RMP** | -92 | -92 | -93 | -93 | -95 |
| **Triangulation** | 107 | 110 | 115 | 128 | 172 |
| **STV** | 0.29 | 0.90 | 0.54 | 2.20 | 5.81 |
| **Alternans** | 0.42 | 1.31 | 0.88 | 3.13 | 9.42 |
| **ERP** | 0.47 | 0.45 | 0.47 | 0.47 | 0.48 |
| **Max Dispersion** | 1.43 | 5.30 | 3.03 | 10.15 | 24.68 |
| **SD** | 28 | 25 | 28 | 35 | 56 |
| **Beat Escape** | 0 | 1 | 1 | 2 | 3 |
| **Refractoriness Escape** | 0 | 0 | 0 | 0 | 0 |
| **EADs** | 0 | 0 | 0 | 0 | 0 |

### 3.2.2: AVERAGE PERCENTAGE CHANGES COMPARED TO VEHICLE CONTROL FOR ACTION POTENTIAL PARAMETERS FOR TERODILINE AT 2Hz ON VENTRICULAR TRABECULAE AS A FUNCTION OF THE MULTIPLE OF FREE ETPC (0.145µM)

| **Parameters** | **2-FOLD** | **21-FOLD** | **69-OLD** | **207-FOLD** |
| --- | --- | --- | --- | --- |
| **APD_30_** | 3 | -2 | -10 | -7 |
| **APD_50_** | 2 | -3 | -9 | -2 |
| **APD_90_** | 3 | 2 | 4 | 25 |
| **AMAX** | 1 | 0 | -3 | -8 |
| **RMP** | 0 | 0 | 0 | 3 |
| **Triangulation** | 3 | 8 | 19 | 60 |
| **STV** | 164 | 165 | 487 | 3983 |
| **Alternans** | 212 | 109 | 646 | 2143 |
| **ERP** | -3 | -1 | -1 | 1 |
| **Max Dispersion** | 272 | 112 | 612 | 1632 |
| **SD** | -13 | -3 | 22 | 95 |
| **Beat Escape** | 25 | 25 | 50 | 75 |
| **Refractoriness Escape** | 0 | 0 | 0 | 0 |
| **EADs** | 0 | 0 | 0 | 0 |

# 4. RESULTS RANOLAZINE

Averaged raw data and averaged percent change data for vehicle control and for ranolazine at 1 and 2 Hz are summarized for each concentration tested in the tables. Data represent the mean of n=4 trabeculae tested in the experimental series.

## **4.1 SUMMARY RANOLAZINE AT 1Hz**

### 4.1.1: AVERAGE RAW DATA OF ACTION POTENTIAL PARAMETERS FOR VEHICLE CONTROL AND RANOLAZINE AT 1Hz ON VENTRICULAR TRABECULAE AS A FUNCTION OF THE MULTIPLE OF FREE ETPC (2.3µM)

| **Parameters** | **Control** | **0.4-FOLD** | **1.3-FOLD** | **13-FOLD** | **43-FOLD** |
| --- | --- | --- | --- | --- | --- |
| **APD_30_** | 144 | 146 | 136 | 126 | 116 |
| **APD_50_** | 195 | 198 | 187 | 181 | 170 |
| **APD_90_** | 264 | 266 | 257 | 274 | 294 |
| **AMAX** | 109 | 112 | 111 | 113 | 109 |
| **RMP** | -94 | -95 | -99 | -96 | -95 |
| **Triangulation** | 121 | 121 | 121 | 148 | 178 |
| **STV** | 0.38 | 0.22 | 0.27 | 0.29 | 0.24 |
| **Alternans** | 0.52 | 0.34 | 0.37 | 0.39 | 0.34 |
| **ERP** | 0.47 | 0.47 | 0.48 | 0.50 | 0.49 |
| **Max Dispersion** | 2.08 | 1.65 | 1.42 | 1.45 | 1.75 |
| **SD** | 46 | 36 | 53 | 39 | 43 |
| **Beat Escape** | 0 | 0 | 0 | 1 | 3 |
| **Refractoriness Escape** | 0 | 0 | 0 | 0 | 0 |
| **EADs** | 0 | 0 | 0 | 0 | 0 |

### 4.1.2: AVERAGE PERCENTAGE CHANGES COMPARED TO VEHICLE CONTROL FOR ACTION POTENTIAL PARAMETERS FOR RANOLAZINE AT 1Hz ON VENTRICULAR TRABECULAE AS A FUNCTION OF THE MULTIPLE OF FREE ETPC (2.3µM)

###

| **Parameters** | **0.4-FOLD** | **1.3-FOLD** | **13-FOLD** | **43-FOLD** |
| --- | --- | --- | --- | --- |
| **APD_30_** | 2 | -5 | -13 | -20 |
| **APD_50_** | 2 | -5 | -7 | -13 |
| **APD_90_** | 1 | -3 | 4 | 12 |
| **AMAX** | 3 | 1 | 3 | 0 |
| **RMP** | 2 | 5 | 2 | 1 |
| **Triangulation** | 1 | 1 | 25 | 52 |
| **STV** | -37 | -21 | -22 | -34 |
| **Alternans** | -35 | -28 | -26 | -35 |
| **ERP** | -1 | 0 | 5 | 4 |
| **Max Dispersion** | -20 | -31 | -30 | -16 |
| **SD** | -22 | 17 | -14 | -6 |
| **Beat Escape** | 0 | 0 | 25 | 75 |
| **Refractoriness Escape** | 0 | 0 | 0 | 0 |
| **EADs** | 0 | 0 | 0 | 0 |

## **4.2 SUMMARY RANOLAZINE AT 2Hz**

### 4.2.1: AVERAGE RAW DATA OF ACTION POTENTIAL PARAMETERS FOR VEHICLE CONTROL AND RANOLAZINE AT 2Hz ON VENTRICULAR TRABECULAE AS A FUNCTION OF THE MULTIPLE OF FREE ETPC (2.3µM)

| **Parameters** | **Control** | **0.4-FOLD** | **1.3-FOLD** | **13-FOLD** | **43-FOLD** |
| --- | --- | --- | --- | --- | --- |
| **APD_30_** | 107 | 108 | 102 | 99 | 104 |
| **APD_50_** | 150 | 151 | 144 | 143 | 151 |
| **APD_90_** | 216 | 215 | 210 | 230 | 275 |
| **AMAX** | 107 | 111 | 111 | 111 | 105 |
| **RMP** | -92 | -94 | -95 | -95 | -95 |
| **Triangulation** | 108 | 107 | 108 | 131 | 172 |
| **STV** | 0.40 | 0.38 | 0.37 | 0.38 | 4.34 |
| **Alternans** | 0.56 | 0.59 | 0.47 | 0.57 | 6.16 |
| **ERP** | 0.48 | 0.48 | 0.49 | 0.49 | 0.47 |
| **Max Dispersion** | 2.35 | 3.13 | 2.18 | 2.23 | 17.83 |
| **SD** | 31 | 22 | 39 | 25 | 25 |
| **Beat Escape** | 0 | 0 | 0 | 0 | 2 |
| **Refractoriness Escape** | 0 | 0 | 0 | 0 | 0 |
| **EADs** | 0 | 0 | 0 | 0 | 0 |

### 4.2.2: AVERAGE PERCENTAGE CHANGES COMPARED TO VEHICLE CONTROL FOR ACTION POTENTIAL PARAMETERS FOR RANOLAZINE AT 2Hz ON VENTRICULAR TRABECULAE AS A FUNCTION OF THE MULTIPLE OF FREE ETPC (2.3µM)

###

| **Parameters** | **0.4-FOLD** | **1.3-FOLD** | **13-FOLD** | **43-FOLD** |
| --- | --- | --- | --- | --- |
| **APD_30_** | 1 | -5 | -7 | -4 |
| **APD_50_** | 1 | -5 | -5 | 1 |
| **APD_90_** | 0 | -3 | 7 | 29 |
| **AMAX** | 3 | 4 | 4 | -2 |
| **RMP** | 2 | 3 | 4 | 3 |
| **Triangulation** | 0 | 0 | 24 | 65 |
| **STV** | -5 | -9 | -3 | 1260 |
| **Alternans** | 4 | -16 | 1 | 990 |
| **ERP** | 0 | 1 | 1 | -2 |
| **Max Dispersion** | 33 | -7 | -5 | 659 |
| **SD** | -27 | 26 | -17 | -18 |
| **Beat Escape** | 0 | 0 | 0 | 50 |
| **Refractoriness Escape** | 0 | 0 | 0 | 0 |
| **EADs** | 0 | 0 | 0 | 0 |

# 5. RESULTS FLECAINIDE

Averaged raw data and averaged percent change data for vehicle control and for flecainide at 1 and 2 Hz are summarized for each concentration tested in the tables. Data represent the mean of n=4 trabeculae tested in the experimental series.

## **5.1 SUMMARY FLECAINIDE AT 1Hz**

### 5.1.1: AVERAGE RAW DATA OF ACTION POTENTIAL PARAMETERS FOR VEHICLE CONTROL AND FLECAINIDE AT 1Hz ON VENTRICULAR TRABECULAE AS A FUNCTION OF THE MULTIPLE OF FREE ETPC (0.75µM)

###

| **Parameters** | **Control** | **1-FOLD** | **13-FOLF** | **40-FOLD** | **133-FOLD** |
| --- | --- | --- | --- | --- | --- |
| **APD_30_** | 205 | 181 | 152 | 125 | 96 |
| **APD_50_** | 268 | 241 | 208 | 172 | 130 |
| **APD_90_** | 338 | 315 | 300 | 278 | 216 |
| **AMAX** | 112 | 108 | 107 | 101 | 80 |
| **RMP** | -88 | -88 | -87 | -88 | -90 |
| **Triangulation** | 133 | 135 | 147 | 153 | 120 |
| **STV** | 0.43 | 0.30 | 1.01 | 8.00 | 7.16 |
| **Alternans** | 0.57 | 0.43 | 1.42 | 11.20 | 6.01 |
| **ERP** | 0.43 | 0.44 | 0.46 | 0.45 | 0.39 |
| **Max Dispersion** | 2.40 | 2.75 | 7.03 | 18.33 | 87.40 |
| **SD** | 41 | 33 | 9 | 24 | 47 |
| **Beat Escape** | 0 | 0 | 1 | 2 | 3 |
| **Refractoriness Escape** | 0 | 0 | 0 | 0 | 0 |
| **EADs** | 0 | 0 | 0 | 0 | 0 |

### 5.1.2: AVERAGE PERCENTAGE CHANGES COMPARED TO VEHICLE CONTROL FOR ACTION POTENTIAL PARAMETERS FOR FLECAINIDE AT 1Hz ON VENTRICULAR TRABECULAE AS A FUNCTION OF THE MULTIPLE OF FREE ETPC (0.75µM)

###

| **Parameters** | **1-FOLD** | **13-FOLF** | **40-FOLD** | **133-FOLD** |
| --- | --- | --- | --- | --- |
| **APD_30_** | -11 | -24 | -38 | -53 |
| **APD_50_** | -10 | -21 | -34 | -52 |
| **APD_90_** | -6 | -10 | -17 | -36 |
| **AMAX** | -3 | -4 | -10 | -29 |
| **RMP** | 0 | -1 | 0 | -2 |
| **Triangulation** | 1 | 10 | 16 | -13 |
| **STV** | 25 | 231 | 2696 | 3601 |
| **Alternans** | -23 | 150 | 1870 | 957 |
| **ERP** | 2 | 7 | 4 | -9 |
| **Max Dispersion** | 15 | 193 | 664 | 3542 |
| **SD** | -19 | -79 | -41 | 14 |
| **Beat Escape** | 0 | 25 | 50 | 75 |
| **Refractoriness Escape** | 0 | 0 | 0 | 0 |
| **EADs** | 0 | 0 | 0 | 0 |

## **5.2 SUMMARY FLECAINIDE AT 2Hz**

### 5.2.1: AVERAGE RAW DATA OF ACTION POTENTIAL PARAMETERS FOR VEHICLE CONTROL AND FLECAINIDE AT 2Hz ON VENTRICULAR TRABECULAE AS A FUNCTION OF THE MULTIPLE OF FREE ETPC (0.75µM)

| **Parameters** | **Control** | **1-FOLD** | **13-FOLF** | **40-FOLD** | **133-FOLD** |
| --- | --- | --- | --- | --- | --- |
| **APD_30_** | 146 | 129 | 121 | 125 | 108 |
| **APD_50_** | 201 | 182 | 164 | 173 | 144 |
| **APD_90_** | 268 | 255 | 252 | 280 | 238 |
| **AMAX** | 112 | 111 | 105 | 97 | 87 |
| **RMP** | -88 | -88 | -89 | -89 | -97 |
| **Triangulation** | 122 | 126 | 131 | 155 | 129 |
| **STV** | 0.88 | 1.25 | 2.88 | 3.51 | 20.03 |
| **Alternans** | 1.26 | 1.72 | 4.13 | 5.07 | 28.04 |
| **ERP** | 0.48 | 0.48 | 0.44 | 0.45 | 0.39 |
| **Max Dispersion** | 3.70 | 6.05 | 10.15 | 13.98 | 88.00 |
| **SD** | 28 | 19 | 7 | 13 | 17 |
| **Beat Escape** | 0 | 0 | 3 | 4 | 4 |
| **Refractoriness Escape** | 0 | 0 | 0 | 0 | 0 |
| **EADs** | 0 | 0 | 0 | 0 | 0 |

### 5.2.2: AVERAGE PERCENTAGE CHANGES COMPARED TO VEHICLE CONTROL FOR ACTION POTENTIAL PARAMETERS FOR FLECAINIDE AT 2Hz ON VENTRICULAR TRABECULAE AS A FUNCTION OF THE MULTIPLE OF FREE ETPC (0.75µM)

| **Parameters** | **1-FOLD** | **13-FOLF** | **40-FOLD** | **133-FOLD** |
| --- | --- | --- | --- | --- |
| **APD_30_** | -11 | -17 | -15 | -27 |
| **APD_50_** | -9 | -17 | -13 | -27 |
| **APD_90_** | -4 | -5 | 5 | -10 |
| **AMAX** | -1 | -6 | -14 | -21 |
| **RMP** | 0 | 1 | 1 | 8 |
| **Triangulation** | 2 | 7 | 29 | 12 |
| **STV** | 55 | 621 | 790 | 5795 |
| **Alternans** | 36 | 227 | 302 | 2122 |
| **ERP** | 1 | -7 | -6 | -19 |
| **Max Dispersion** | 64 | 174 | 278 | 2278 |
| **SD** | -32 | -74 | -54 | -41 |
| **Beat Escape** | 0 | 75 | 100 | 100 |
| **Refractoriness Escape** | 0 | 0 | 0 | 0 |
| **EADs** | 0 | 0 | 0 | 0 |

# 6. RESULTS SERTINDOLE

Averaged raw data and averaged percent change data for vehicle control and for sertindole at 1 and 2 Hz are summarized for each concentration tested in the tables. Data represent the mean of n=4 trabeculae tested in the experimental series.

## **6.1 SUMMARY SERTINDOLE AT 1Hz**

### 6.1.1: AVERAGE RAW DATA OF ACTION POTENTIAL PARAMETERS FOR VEHICLE CONTROL AND SERTINDOLE AT 1Hz ON VENTRICULAR TRABECULAE AS A FUNCTION OF THE MULTIPLE OF FREE ETPC (0.002µM)

###

| **Parameters** | **Control** | **1.5-FOLD** | **15-FOLD** | **150-FOLD** | **1500-FOLD** |
| --- | --- | --- | --- | --- | --- |
| **APD_30_** | 152 | 159 | 150 | 158 | 153 |
| **APD_50_** | 196 | 205 | 195 | 207 | 206 |
| **APD_90_** | 260 | 272 | 261 | 279 | 282 |
| **AMAX** | 107 | 112 | 112 | 112 | 111 |
| **RMP** | -87 | -88 | -89 | -89 | -89 |
| **Triangulation** | 108 | 113 | 111 | 121 | 129 |
| **STV** | 0.40 | 0.54 | 0.39 | 0.47 | 0.31 |
| **Alternans** | 0.54 | 0.83 | 0.51 | 0.71 | 0.47 |
| **ERP** | 0.41 | 0.41 | 0.42 | 0.44 | 0.46 |
| **Max Dispersion** | 3.68 | 3.13 | 2.38 | 3.55 | 2.58 |
| **SD (APD_90_)** | 36 | 31 | 29 | 40 | 38 |
| **Beat Escape** | 0 | 0 | 0 | 0 | 0 |
| **Refractoriness Escape** | 0 | 0 | 0 | 0 | 0 |
| **EADs** | 0 | 0 | 0 | 0 | 0 |

### 6.1.2: AVERAGE PERCENTAGE CHANGES COMPARED TO VEHICLE CONTROL FOR ACTION POTENTIAL PARAMETERS FOR SERTINDOLE AT 1Hz ON VENTRICULAR TRABECULAE AS A FUNCTION OF THE MULTIPLE OF FREE ETPC (0.002µM)

###

| **Parameters** | **1.5-FOLD** | **15-FOLD** | **150-FOLD** | **1500-FOLD** |
| --- | --- | --- | --- | --- |
| **APD_30_** | 6 | -1 | 4 | 1 |
| **APD_50_** | 6 | 0 | 6 | 6 |
| **APD_90_** | 5 | 0 | 7 | 8 |
| **AMAX** | 5 | 5 | 5 | 4 |
| **RMP** | 1 | 3 | 2 | 3 |
| **Triangulation** | 4 | 3 | 12 | 19 |
| **STV** | 31 | 0 | 23 | -24 |
| **Alternans** | 54 | -5 | 31 | -12 |
| **ERP** | 1 | 3 | 7 | 13 |
| **Max Dispersion** | -15 | -35 | -3 | -30 |
| **SD** | -14 | -20 | 10 | 5 |
| **Beat Escape** | 0 | 0 | 0 | 0 |
| **Refractoriness Escape** | 0 | 0 | 0 | 0 |
| **EADs** | 0 | 0 | 0 | 0 |

## **6.2 SUMMARY SERTINDOLE AT 2Hz**

### 6.2.1: AVERAGE RAW DATA OF ACTION POTENTIAL PARAMETERS FOR VEHICLE CONTROL AND SERTINDOLE AT 2Hz ON VENTRICULAR TRABECULAE AS A FUNCTION OF THE MULTIPLE OF FREE ETPC (0.002µM)

| **Parameters** | **Control** | **1.5-FOLD** | **15-FOLD** | **150-FOLD** | **1500-FOLD** |
| --- | --- | --- | --- | --- | --- |
| **APD_30_** | 106 | 104 | 104 | 112 | 107 |
| **APD_50_** | 139 | 139 | 139 | 150 | 150 |
| **APD_90_** | 197 | 197 | 199 | 214 | 221 |
| **AMAX** | 106 | 109 | 110 | 110 | 110 |
| **RMP** | -85 | -85 | -87 | -87 | -87 |
| **Triangulation** | 92 | 93 | 95 | 103 | 114 |
| **STV** | 0.61 | 1.20 | 0.48 | 0.78 | 0.42 |
| **Alternans** | 0.83 | 1.61 | 0.69 | 1.08 | 0.59 |
| **ERP** | 0.43 | 0.43 | 0.44 | 0.45 | 0.48 |
| **Max Dispersion** | 4.58 | 4.87 | 3.50 | 3.78 | 2.80 |
| **SD (APD_90_)** | 14 | 9 | 17 | 19 | 23 |
| **Beat Escape** | 0 | 0 | 0 | 0 | 0 |
| **Refractoriness Escape** | 0 | 0 | 0 | 0 | 0 |
| **EADs** | 0 | 0 | 0 | 0 | 0 |

### 6.2.2: AVERAGE PERCENTAGE CHANGES COMPARED TO VEHICLE CONTROL FOR ACTION POTENTIAL PARAMETERS FOR SERTINDOLE AT 2Hz ON VENTRICULAR TRABECULAE AS A FUNCTION OF THE MULTIPLE OF FREE ETPC (0.002µM)

| **Parameters** | **1.5-FOLD** | **15-FOLD** | **150-FOLD** | **1500-FOLD** |
| --- | --- | --- | --- | --- |
| **APD_30_** | -1 | -1 | 6 | 1 |
| **APD_50_** | 0 | 0 | 8 | 8 |
| **APD_90_** | 0 | 1 | 9 | 12 |
| **AMAX** | 3 | 4 | 5 | 4 |
| **RMP** | 1 | 3 | 3 | 3 |
| **Triangulation** | 1 | 3 | 12 | 23 |
| **STV** | 98 | -18 | 29 | -32 |
| **Alternans** | 93 | -17 | 30 | -29 |
| **ERP** | 2 | 4 | 7 | 13 |
| **Max Dispersion** | 7 | -23 | -17 | -39 |
| **SD** | -38 | 17 | 32 | 59 |
| **Beat Escape** | 0 | 0 | 0 | 0 |
| **Refractoriness Escape** | 0 | 0 | 0 | 0 |
| **EADs** | 0 | 0 | 0 | 0 |

##

# 7. RESULTS CISAPRIDE

Averaged raw data and averaged percent change data for vehicle control and for cisapride at 1 and 2 Hz are summarized for each concentration tested in the tables. Data represent the mean of n=4 trabeculae tested in the experimental series.

## **7.1 SUMMARY CISAPRIDE AT 1Hz**

### 7.1.1: AVERAGE RAW DATA OF ACTION POTENTIAL PARAMETERS FOR VEHICLE CONTROL AND CISAPRIDE AT 1Hz ON VENTRICULAR TRABECULAE AS A FUNCTION OF THE MULTIPLE OF FREE ETPC (0.003µM)

###

| **Parameters** | **Control** | **0.1-FOLD** | **1-FOLD** | **10-FOLD** | **100-FOLD** |
| --- | --- | --- | --- | --- | --- |
| **APD_30_** | 180 | 153 | 161 | 148 | 158 |
| **APD_50_** | 236 | 205 | 213 | 206 | 224 |
| **APD_90_** | 311 | 281 | 286 | 285 | 312 |
| **AMAX** | 113 | 111 | 111 | 111 | 116 |
| **RMP** | -95 | -94 | -96 | -99 | -102 |
| **Triangulation** | 132 | 127 | 124 | 137 | 154 |
| **STV** | 0.44 | 0.54 | 0.57 | 0.27 | 0.37 |
| **Alternans** | 0.65 | 0.75 | 0.76 | 0.37 | 0.54 |
| **ERP** | 0.43 | 0.45 | 0.44 | 0.48 | 0.49 |
| **Max Dispersion** | 2.95 | 3.28 | 3.20 | 2.73 | 2.75 |
| **SD (APD_90_)** | 50 | 48 | 64 | 46 | 75 |
| **Beat Escape** | 0 | 0 | 0 | 0 | 0 |
| **Refractoriness Escape** | 0 | 0 | 0 | 0 | 0 |
| **EADs** | 0 | 0 | 0 | 0 | 0 |

### 7.1.2: AVERAGE PERCENTAGE CHANGES COMPARED TO VEHICLE CONTROL FOR ACTION POTENTIAL PARAMETERS FOR CISAPRIDE AT 1Hz ON VENTRICULAR TRABECULAE AS A FUNCTION OF THE MULTIPLE OF FREE ETPC (0.003µM)

###

| **Parameters** | **0.1-FOLD** | **1-FOLD** | **10-FOLD** | **100-FOLD** |
| --- | --- | --- | --- | --- |
| **APD_30_** | -14 | -11 | -17 | -12 |
| **APD_50_** | -13 | -10 | -13 | -6 |
| **APD_90_** | -10 | -9 | -8 | 0 |
| **AMAX** | -2 | -2 | -1 | 3 |
| **RMP** | -1 | 0 | 4 | 7 |
| **Triangulation** | -3 | -5 | 4 | 17 |
| **STV** | 22 | 42 | -31 | 3 |
| **Alternans** | 14 | 16 | -44 | -17 |
| **ERP** | 3 | 1 | 11 | 14 |
| **Max Dispersion** | 11 | 8 | -8 | -7 |
| **SD** | -3 | 29 | -8 | 50 |
| **Beat Escape** | 0 | 0 | 0 | 0 |
| **Refractoriness Escape** | 0 | 0 | 0 | 0 |
| **EADs** | 0 | 0 | 0 | 0 |

## **7.2 SUMMARY CISAPRIDE AT 2Hz**

### 7.2.1: AVERAGE RAW DATA OF ACTION POTENTIAL PARAMETERS FOR VEHICLE CONTROL AND CISAPRIDE AT 2Hz ON VENTRICULAR TRABECULAE AS A FUNCTION OF THE MULTIPLE OF FREE ETPC (0.003µM)

| **Parameters** | **Control** | **0.1-FOLD** | **1-FOLD** | **10-FOLD** | **100-FOLD** |
| --- | --- | --- | --- | --- | --- |
| **APD_30_** | 126 | 114 | 115 | 110 | 114 |
| **APD_50_** | 172 | 157 | 156 | 157 | 168 |
| **APD_90_** | 243 | 228 | 225 | 231 | 254 |
| **AMAX** | 112 | 110 | 109 | 114 | 114 |
| **RMP** | -95 | -94 | -94 | -100 | -101 |
| **Triangulation** | 117 | 114 | 110 | 122 | 140 |
| **STV** | 0.56 | 0.76 | 0.66 | 0.45 | 0.56 |
| **Alternans** | 0.83 | 0.98 | 0.99 | 0.63 | 0.75 |
| **ERP** | 0.46 | 0.47 | 0.46 | 0.50 | 0.52 |
| **Max Dispersion** | 3.97 | 4.08 | 4.38 | 2.55 | 3.75 |
| **SD (APD_90_)** | 39 | 34 | 47 | 43 | 58 |
| **Beat Escape** | 0 | 0 | 0 | 0 | 0 |
| **Refractoriness Escape** | 0 | 0 | 0 | 0 | 0 |
| **EADs** | 0 | 0 | 0 | 0 | 0 |

### 7.2.2: AVERAGE PERCENTAGE CHANGES COMPARED TO VEHICLE CONTROL FOR ACTION POTENTIAL PARAMETERS FOR CISAPRIDE AT 2Hz ON VENTRICULAR TRABECULAE AS A FUNCTION OF THE MULTIPLE OF FREE ETPC (0.003µM)

| **Parameters** | **0.1-FOLD** | **1-FOLD** | **10-FOLD** | **100-FOLD** |
| --- | --- | --- | --- | --- |
| **APD_30_** | -10 | -10 | -13 | -10 |
| **APD_50_** | -8 | -10 | -9 | -3 |
| **APD_90_** | -6 | -8 | -5 | 4 |
| **AMAX** | -2 | -3 | 1 | 2 |
| **RMP** | 0 | 0 | 6 | 7 |
| **Triangulation** | -2 | -6 | 4 | 20 |
| **STV** | 443 | 282 | 155 | 214 |
| **Alternans** | 18 | 20 | -24 | -9 |
| **ERP** | 1 | 1 | 10 | 13 |
| **Max Dispersion** | 3 | 10 | -36 | -6 |
| **SD** | -11 | 22 | 11 | 49 |
| **Beat Escape** | 0 | 0 | 0 | 0 |
| **Refractoriness Escape** | 0 | 0 | 0 | 0 |
| **EADs** | 0 | 0 | 0 | 0 |

# 8. RESULTS TERFENADINE

Averaged raw data and averaged percent change data for vehicle control and for terfenadine at 1 and 2 Hz are summarized for each concentration tested in the tables. Data represent the mean of n=4 trabeculae tested in the experimental series.

## **8.1 SUMMARY TERFENADINE AT 1Hz**

### 8.1.1: AVERAGE RAW DATA OF ACTION POTENTIAL PARAMETERS FOR VEHICLE CONTROL AND TERFENADINE AT 1Hz ON VENTRICULAR TRABECULAE AS A FUNCTION OF THE MULTIPLE OF FREE ETPC (0.009µM)

###

| **Parameters** | **Control** | **1-FOLD** | **11-FOLD** | **111-FOLD** | **1111-FOLD** |
| --- | --- | --- | --- | --- | --- |
| **APD_30_** | 143 | 148 | 152 | 148 | 140 |
| **APD_50_** | 195 | 199 | 203 | 200 | 192 |
| **APD_90_** | 277 | 278 | 286 | 284 | 277 |
| **AMAX** | 111 | 113 | 109 | 109 | 110 |
| **RMP** | -88 | -89 | -86 | -85 | -87 |
| **Triangulation** | 135 | 130 | 134 | 135 | 137 |
| **STV** | 0.32 | 0.50 | 0.45 | 0.39 | 0.44 |
| **Alternans** | 0.46 | 0.70 | 0.62 | 0.52 | 0.60 |
| **ERP** | 0.45 | 0.45 | 0.44 | 0.45 | 0.46 |
| **Max Dispersion** | 2.80 | 4.48 | 2.80 | 2.43 | 3.70 |
| **SD (APD_90_)** | 65 | 59 | 68 | 71 | 70 |
| **Beat Escape** | 0 | 0 | 0 | 0 | 0 |
| **Refractoriness Escape** | 0 | 0 | 0 | 0 | 0 |
| **EADs** | 0 | 0 | 0 | 0 | 0 |

### 8.1.2: AVERAGE PERCENTAGE CHANGES COMPARED TO VEHICLE CONTROL FOR ACTION POTENTIAL PARAMETERS FOR TERFENADINE AT 1Hz ON VENTRICULAR TRABECULAE AS A FUNCTION OF THE MULTIPLE OF FREE ETPC (0.009µM)

###

| **Parameters** | **1-FOLD** | **11-FOLD** | **111-FOLD** | **1111-FOLD** |
| --- | --- | --- | --- | --- |
| **APD_30_** | 4 | 6 | 4 | -2 |
| **APD_50_** | 3 | 4 | 3 | -2 |
| **APD_90_** | 1 | 3 | 2 | 0 |
| **AMAX** | 1 | -1 | -2 | 0 |
| **RMP** | 1 | -2 | -3 | -1 |
| **Triangulation** | -2 | 0 | 0 | 2 |
| **STV** | 72 | 40 | 30 | 56 |
| **Alternans** | 53 | 35 | 12 | 31 |
| **ERP** | -2 | -3 | 0 | 2 |
| **Max Dispersion** | 60 | 0 | -13 | 32 |
| **SD** | -10 | 4 | 9 | 8 |
| **Beat Escape** | 0 | 0 | 0 | 0 |
| **Refractoriness Escape** | 0 | 0 | 0 | 0 |
| **EADs** | 0 | 0 | 0 | 0 |

## **8.2 SUMMARY TERFENADINE AT 2Hz**

### 8.2.1: AVERAGE RAW DATA OF ACTION POTENTIAL PARAMETERS FOR VEHICLE CONTROL AND TERFENADINE AT 2Hz ON VENTRICULAR TRABECULAE AS A FUNCTION OF THE MULTIPLE OF FREE ETPC (0.009µM)

| **Parameters** | **Control** | **1-FOLD** | **11-FOLD** | **111-FOLD** | **1111-FOLD** |
| --- | --- | --- | --- | --- | --- |
| **APD_30_** | 101 | 107 | 110 | 108 | 105 |
| **APD_50_** | 145 | 151 | 153 | 154 | 150 |
| **APD_90_** | 225 | 228 | 231 | 234 | 233 |
| **AMAX** | 106 | 109 | 108 | 109 | 108 |
| **RMP** | -87 | -87 | -86 | -85 | -85 |
| **Triangulation** | 125 | 120 | 121 | 126 | 128 |
| **STV** | 0.73 | 0.91 | 0.66 | 0.89 | 0.71 |
| **Alternans** | 1.06 | 1.31 | 0.97 | 1.28 | 0.96 |
| **ERP** | 0.50 | 0.48 | 0.47 | 0.48 | 0.48 |
| **Max Dispersion** | 4.92 | 5.60 | 4.40 | 5.53 | 3.10 |
| **SD (APD_90_)** | 59 | 47 | 48 | 57 | 60 |
| **Beat Escape** | 0 | 0 | 0 | 0 | 0 |
| **Refractoriness Escape** | 0 | 0 | 0 | 0 | 0 |
| **EADs** | 0 | 0 | 0 | 0 | 0 |

### 8.2.2: AVERAGE PERCENTAGE CHANGES COMPARED TO VEHICLE CONTROL FOR ACTION POTENTIAL PARAMETERS FOR TERFENADINE AT 2Hz ON VENTRICULAR TRABECULAE AS A FUNCTION OF THE MULTIPLE OF FREE ETPC (0.009µM)

| **Parameters** | **1-FOLD** | **11-FOLD** | **111-FOLD** | **1111-FOLD** |
| --- | --- | --- | --- | --- |
| **APD_30_** | 8 | 11 | 10 | 5 |
| **APD_50_** | 6 | 7 | 7 | 4 |
| **APD_90_** | 2 | 4 | 4 | 3 |
| **AMAX** | 2 | 2 | 2 | 2 |
| **RMP** | 1 | -1 | -2 | -2 |
| **Triangulation** | -2 | -2 | 0 | 3 |
| **STV** | 56 | 7 | 26 | -2 |
| **Alternans** | 24 | -8 | 21 | -10 |
| **ERP** | -3 | -6 | -2 | -3 |
| **Max Dispersion** | 14 | -11 | 12 | -37 |
| **SD** | -20 | -18 | -3 | 3 |
| **Beat Escape** | 0 | 0 | 0 | 0 |
| **Refractoriness Escape** | 0 | 0 | 0 | 0 |
| **EADs** | 0 | 0 | 0 | 0 |

# 9. RESULTS ALFUZOSIN

Averaged raw data and averaged percent change data for vehicle control and for alfuzosin at 1 and 2 Hz are summarized for each concentration tested in the tables. Data represent the mean of n=4 trabeculae tested in the experimental series.

## **9.1 SUMMARY ALFUZOSIN AT 1Hz**

### 9.1.1: AVERAGE RAW DATA OF ACTION POTENTIAL PARAMETERS FOR VEHICLE CONTROL AND ALFUZOSIN AT 1Hz ON VENTRICULAR TRABECULAE AS A FUNCTION OF THE MULTIPLE OF FREE ETPC (0.006µM)

###

| **Parameters** | **Control** | **1.6-FOLD** | **17-FOLD** | **167-FOLD** | **1667-FOLD** |
| --- | --- | --- | --- | --- | --- |
| **APD_30_** | 156 | 163 | 159 | 161 | 162 |
| **APD_50_** | 204 | 214 | 209 | 213 | 222 |
| **APD_90_** | 280 | 287 | 284 | 291 | 319 |
| **AMAX** | 108 | 110 | 110 | 110 | 109 |
| **RMP** | -85 | -88 | -88 | -89 | -88 |
| **Triangulation** | 124 | 123 | 126 | 130 | 158 |
| **STV** | 0.29 | 0.30 | 0.26 | 0.29 | 0.46 |
| **Alternans** | 0.39 | 0.33 | 0.34 | 0.45 | 0.72 |
| **ERP** | 0.42 | 0.42 | 0.44 | 0.44 | 0.47 |
| **Max Dispersion** | 1.88 | 2.40 | 2.18 | 2.43 | 3.05 |
| **SD (APD_90_)** | 18 | 21 | 28 | 14 | 13 |
| **Beat Escape** | 0 | 0 | 0 | 0 | 0 |
| **Refractoriness Escape** | 0 | 0 | 0 | 0 | 0 |
| **EADs** | 0 | 0 | 0 | 0 | 0 |

### 9.1.2: AVERAGE PERCENTAGE CHANGES COMPARED TO VEHICLE CONTROL FOR ACTION POTENTIAL PARAMETERS FOR ALFUZOSIN AT 1Hz ON VENTRICULAR TRABECULAE AS A FUNCTION OF THE MULTIPLE OF FREE ETPC (0.006µM)

###

| **Parameters** | **1.6-FOLD** | **17-FOLD** | **167-FOLD** | **1667-FOLD** |
| --- | --- | --- | --- | --- |
| **APD_30_** | 4 | 2 | 3 | 4 |
| **APD_50_** | 4 | 2 | 4 | 9 |
| **APD_90_** | 3 | 1 | 4 | 14 |
| **AMAX** | 2 | 1 | 2 | 1 |
| **RMP** | 3 | 3 | 4 | 4 |
| **Triangulation** | -1 | 1 | 5 | 28 |
| **STV** | 9 | -6 | 7 | 75 |
| **Alternans** | -14 | -13 | 16 | 86 |
| **ERP** | 0 | 4 | 4 | 12 |
| **Max Dispersion** | 28 | 16 | 29 | 63 |
| **SD** | 17 | 57 | -22 | -29 |
| **Beat Escape** | 0 | 0 | 0 | 0 |
| **Refractoriness Escape** | 0 | 0 | 0 | 0 |
| **EADs** | 0 | 0 | 0 | 0 |

## **9.2 SUMMARY ALFUZOSIN AT 2Hz**

### 9.2.1: AVERAGE RAW DATA OF ACTION POTENTIAL PARAMETERS FOR VEHICLE CONTROL AND ALFUZOSIN AT 2Hz ON VENTRICULAR TRABECULAE AS A FUNCTION OF THE MULTIPLE OF FREE ETPC (0.006µM)

| **Parameters** | **Control** | **1.6-FOLD** | **17-FOLD** | **167-FOLD** | **1667-FOLD** |
| --- | --- | --- | --- | --- | --- |
| **APD_30_** | 117 | 120 | 116 | 115 | 115 |
| **APD_50_** | 158 | 163 | 160 | 161 | 165 |
| **APD_90_** | 231 | 234 | 233 | 238 | 258 |
| **AMAX** | 108 | 109 | 110 | 110 | 108 |
| **RMP** | -85 | -87 | -88 | -88 | -87 |
| **Triangulation** | 114 | 115 | 118 | 123 | 142 |
| **STV** | 0.61 | 0.64 | 0.66 | 0.63 | 0.55 |
| **Alternans** | 0.87 | 0.92 | 0.99 | 0.83 | 0.78 |
| **ERP** | 0.45 | 0.45 | 0.47 | 0.48 | 0.50 |
| **Max Dispersion** | 3.53 | 3.95 | 3.93 | 2.88 | 2.72 |
| **SD (APD_90_)** | 14 | 6 | 16 | 10 | 11 |
| **Beat Escape** | 0 | 0 | 0 | 0 | 0 |
| **Refractoriness Escape** | 0 | 0 | 0 | 0 | 0 |
| **EADs** | 0 | 0 | 0 | 0 | 0 |

### 9.2.2: AVERAGE PERCENTAGE CHANGES COMPARED TO VEHICLE CONTROL FOR ACTION POTENTIAL PARAMETERS FOR ALFUZOSIN AT 2Hz ON VENTRICULAR TRABECULAE AS A FUNCTION OF THE MULTIPLE OF FREE ETPC (0.006µM)

| **Parameters** | **1.6-FOLD** | **17-FOLD** | **167-FOLD** | **1667-FOLD** |
| --- | --- | --- | --- | --- |
| **APD_30_** | 2 | -1 | -1 | -1 |
| **APD_50_** | 3 | 1 | 2 | 5 |
| **APD_90_** | 2 | 1 | 3 | 12 |
| **AMAX** | 1 | 2 | 2 | 0 |
| **RMP** | 2 | 4 | 3 | 2 |
| **Triangulation** | 1 | 3 | 8 | 26 |
| **STV** | 8 | 13 | 10 | -4 |
| **Alternans** | 6 | 14 | -4 | -10 |
| **ERP** | 1 | 4 | 7 | 11 |
| **Max Dispersion** | 12 | 11 | -18 | -23 |
| **SD** | -55 | 12 | -28 | -23 |
| **Beat Escape** | 0 | 0 | 0 | 0 |
| **Refractoriness Escape** | 0 | 0 | 0 | 0 |
| **EADs** | 0 | 0 | 0 | 0 |

# 10. RESULTS SOTALOL (D,L)

Averaged raw data and averaged percent change data for vehicle control and for sotalol (D,L) at 1 and 2 Hz are summarized for each concentration tested in the tables. Data represent the mean of n=4 trabeculae tested in the experimental series.

## **10.1 SUMMARY SOTALOL (D,L) AT 1Hz**

### 10.1.1: AVERAGE RAW DATA OF ACTION POTENTIAL PARAMETERS FOR VEHICLE CONTROL AND SOTALOL (D,L) AT 1Hz ON VENTRICULAR TRABECULAE AS A FUNCTION OF THE MULTIPLE OF FREE ETPC (14.69µM)

###

| **Parameters** | **Control** | **0.2-FOLD** | **0.7-FOLD** | **2-FOLD** | **7-FOLD** |
| --- | --- | --- | --- | --- | --- |
| **APD_30_** | 157 | 154 | 160 | 163 | 175 |
| **APD_50_** | 209 | 214 | 223 | 237 | 267 |
| **APD_90_** | 298 | 302 | 319 | 346 | 408 |
| **AMAX** | 110 | 109 | 108 | 111 | 109 |
| **RMP** | -85 | -84 | -84 | -85 | -85 |
| **Triangulation** | 141 | 148 | 159 | 184 | 233 |
| **STV** | 0.65 | 0.23 | 0.39 | 0.26 | 0.44 |
| **Alternans** | 1.00 | 0.34 | 0.54 | 0.38 | 0.66 |
| **ERP** | 0.43 | 0.48 | 0.48 | 0.52 | 0.55 |
| **Max Dispersion** | 2.85 | 1.88 | 2.85 | 2.33 | 3.47 |
| **SD (APD_90_)** | 10 | 19 | 16 | 23 | 34 |
| **Beat Escape** | 0 | 0 | 0 | 0 | 0 |
| **Refractoriness Escape** | 0 | 0 | 0 | 0 | 0 |
| **EADs** | 0 | 0 | 0 | 0 | 0 |

### 10.1.2: AVERAGE PERCENTAGE CHANGES COMPARED TO VEHICLE CONTROL FOR ACTION POTENTIAL PARAMETERS FOR SOTALOL (D,L) AT 1Hz ON VENTRICULAR TRABECULAE AS A FUNCTION OF THE MULTIPLE OF FREE ETPC (14.69µM)

| **Parameters** | **0.2-FOLD** | **0.7-FOLD** | **2-FOLD** | **7-FOLD** |
| --- | --- | --- | --- | --- |
| **APD_30_** | -2 | 2 | 4 | 11 |
| **APD_50_** | 2 | 7 | 13 | 28 |
| **APD_90_** | 1 | 7 | 16 | 37 |
| **AMAX** | -1 | -1 | 1 | -1 |
| **RMP** | -1 | 0 | 1 | 1 |
| **Triangulation** | 6 | 14 | 32 | 69 |
| **STV** | -50 | -9 | -48 | -10 |
| **Alternans** | -66 | -46 | -62 | -34 |
| **ERP** | 10 | 12 | 19 | 28 |
| **Max Dispersion** | -34 | 0 | -18 | 22 |
| **SD** | 89 | 65 | 136 | 240 |
| **Beat Escape** | 0 | 0 | 0 | 0 |
| **Refractoriness Escape** | 0 | 0 | 0 | 0 |
| **EADs** | 0 | 0 | 0 | 0 |

###

## **10.2 SUMMARY SOTALOL (D,L) AT 2Hz**

### 10.2.1: AVERAGE RAW DATA OF ACTION POTENTIAL PARAMETERS FOR VEHICLE CONTROL AND SOTALOL (D,L) AT 2Hz ON VENTRICULAR TRABECULAE AS A FUNCTION OF THE MULTIPLE OF FREE ETPC (14.69µM)

| **Parameters** | **Control** | **0.2-FOLD** | **0.7-FOLD** | **2-FOLD** | **7-FOLD** |
| --- | --- | --- | --- | --- | --- |
| **APD_30_** | 117 | 117 | 120 | 120 | 125 |
| **APD_50_** | 161 | 166 | 172 | 181 | 198 |
| **APD_90_** | 248 | 252 | 266 | 289 | 337 |
| **AMAX** | 108 | 108 | 105 | 107 | 104 |
| **RMP** | -84 | -83 | -84 | -84 | -82 |
| **Triangulation** | 131 | 136 | 147 | 169 | 211 |
| **STV** | 0.99 | 0.49 | 0.64 | 0.63 | 1.22 |
| **Alternans** | 1.44 | 0.74 | 0.89 | 0.85 | 1.76 |
| **ERP** | 0.45 | 0.48 | 0.50 | 0.53 | 0.55 |
| **Max Dispersion** | 7.12 | 2.85 | 4.15 | 4.22 | 6.45 |
| **SD (APD_90_)** | 13 | 14 | 12 | 17 | 21 |
| **Beat Escape** | 0 | 0 | 0 | 0 | 0 |
| **Refractoriness Escape** | 0 | 0 | 0 | 0 | 0 |
| **EADs** | 0 | 0 | 0 | 0 | 0 |

### 10.2.2: AVERAGE PERCENTAGE CHANGES COMPARED TO VEHICLE CONTROL FOR ACTION POTENTIAL PARAMETERS FOR SOTALOL (D,L) AT 2Hz ON VENTRICULAR TRABECULAE AS A FUNCTION OF THE MULTIPLE OF FREE ETPC (14.69µM)

| **Parameters** | **0.2-FOLD** | **0.7-FOLD** | **2-FOLD** | **7-FOLD** |
| --- | --- | --- | --- | --- |
| **APD_30_** | 0 | 2 | 2 | 7 |
| **APD_50_** | 3 | 7 | 12 | 23 |
| **APD_90_** | 2 | 7 | 17 | 36 |
| **AMAX** | 0 | -3 | -1 | -4 |
| **RMP** | -1 | -1 | -1 | -2 |
| **Triangulation** | 4 | 13 | 32 | 65 |
| **STV** | -48 | -30 | -28 | 49 |
| **Alternans** | -48 | -38 | -41 | 22 |
| **ERP** | 7 | 10 | 16 | 22 |
| **Max Dispersion** | -60 | -42 | -41 | -9 |
| **SD** | 2 | -13 | 25 | 56 |
| **Beat Escape** | 0 | 0 | 0 | 0 |
| **Refractoriness Escape** | 0 | 0 | 0 | 0 |
| **EADs** | 0 | 0 | 0 | 0 |

# 11. RESULTS MOXIFLOXACIN

Averaged raw data and averaged percent change data for vehicle control and for moxifloxacin at 1 and 2 Hz are summarized for each concentration tested in the tables. Data represent the mean of n=4 trabeculae tested in the experimental series.

## **11.1 SUMMARY MOXIFLOXACIN AT 1Hz**

### 11.1.1: AVERAGE RAW DATA OF ACTION POTENTIAL PARAMETERS FOR VEHICLE CONTROL AND MOXIFLOXACIN AT 1Hz ON VENTRICULAR TRABECULAE AS A FUNCTION OF THE MULTIPLE OF FREE ETPC (10.96µM)

###

| **Parameters** | **Control** | **0.9-FOLD** | **3-FOLD** | **9-FOLD** | **91-FOLD** |
| --- | --- | --- | --- | --- | --- |
| **APD_30_** | 183 | 186 | 194 | 213 | 272 |
| **APD_50_** | 237 | 251 | 261 | 296 | 395 |
| **APD_90_** | 314 | 334 | 347 | 403 | 609 |
| **AMAX** | 117 | 116 | 117 | 117 | 99 |
| **RMP** | -89 | -87 | -94 | -90 | -84 |
| **Triangulation** | 131 | 148 | 153 | 191 | 338 |
| **STV** | 0.53 | 0.35 | 0.33 | 0.37 | 33.33 |
| **Alternans** | 0.73 | 0.51 | 0.47 | 0.60 | 47.24 |
| **ERP** | 0.41 | 0.46 | 0.46 | 0.48 | 0.51 |
| **Max Dispersion** | 4.58 | 2.40 | 2.00 | 3.30 | 66.98 |
| **SD (APD_90_)** | 12 | 29 | 27 | 15 | 64 |
| **Beat Escape** | 0 | 0 | 0 | 0 | 0 |
| **Refractoriness Escape** | 0 | 0 | 0 | 0 | 1 |
| **EADs** | 0 | 0 | 0 | 0 | 1 |

### 11.1.2: AVERAGE PERCENTAGE CHANGES COMPARED TO VEHICLE CONTROL FOR ACTION POTENTIAL PARAMETERS FOR MOXIFLOXACIN AT 1Hz ON VENTRICULAR TRABECULAE AS A FUNCTION OF THE MULTIPLE OF FREE ETPC (10.96µM)

| **Parameters** | **0.9-FOLD** | **3-FOLD** | **9-FOLD** | **91-FOLD** |
| --- | --- | --- | --- | --- |
| **APD_30_** | 3 | 7 | 17 | 46 |
| **APD_50_** | 6 | 10 | 25 | 65 |
| **APD_90_** | 6 | 11 | 28 | 94 |
| **AMAX** | -1 | 0 | 0 | -16 |
| **RMP** | -1 | 6 | 1 | -6 |
| **Triangulation** | 13 | 17 | 47 | 164 |
| **STV** | -31 | -27 | -19 | 6257 |
| **Alternans** | -31 | -36 | -18 | 6327 |
| **ERP** | 11 | 10 | 17 | 24 |
| **Max Dispersion** | -48 | -56 | -28 | 1364 |
| **SD** | 149 | 129 | 30 | 447 |
| **Beat Escape** | 0 | 0 | 0 | 0 |
| **Refractoriness Escape** | 0 | 0 | 0 | 25 |
| **EADs** | 0 | 0 | 0 | 25 |

###

## **11.2 SUMMARY MOXIFLOXACIN AT 2Hz**

### 11.2.1: AVERAGE RAW DATA OF ACTION POTENTIAL PARAMETERS FOR VEHICLE CONTROL AND MOXIFLOXACIN AT 2Hz ON VENTRICULAR TRABECULAE AS A FUNCTION OF THE MULTIPLE OF FREE ETPC (10.96µM)

| **Parameters** | **Control** | **0.9-FOLD** | **3-FOLD** | **9-FOLD** | **91-FOLD** |
| --- | --- | --- | --- | --- | --- |
| **APD_30_** | 128 | 134 | 139 | 148 | 283 |
| **APD_50_** | 174 | 183 | 191 | 208 | 385 |
| **APD_90_** | 247 | 261 | 273 | 305 | 631 |
| **AMAX** | 116 | 114 | 116 | 115 | 93 |
| **RMP** | -89 | -89 | -96 | -92 | -88 |
| **Triangulation** | 119 | 128 | 134 | 157 | 347 |
| **STV** | 0.59 | 0.76 | 0.74 | 0.93 | 8.41 |
| **Alternans** | 0.84 | 1.13 | 1.04 | 1.39 | 11.76 |
| **ERP** | 0.45 | 0.46 | 0.46 | 0.48 | 0.45 |
| **Max Dispersion** | 3.70 | 5.30 | 3.55 | 4.97 | 40.88 |
| **SD (APD_90_)** | 23 | 19 | 13 | 13 | 45 |
| **Beat Escape** | 0 | 0 | 0 | 0 | 1 |
| **Refractoriness Escape** | 0 | 0 | 0 | 0 | 3 |
| **EADs** | 0 | 0 | 0 | 0 | 0 |

### 11.2.2: AVERAGE PERCENTAGE CHANGES COMPARED TO VEHICLE CONTROL FOR ACTION POTENTIAL PARAMETERS FOR MOXIFLOXACIN AT 2Hz ON VENTRICULAR TRABECULAE AS A FUNCTION OF THE MULTIPLE OF FREE ETPC (10.96µM)

| **Parameters** | **0.9-FOLD** | **3-FOLD** | **9-FOLD** | **91-FOLD** |
| --- | --- | --- | --- | --- |
| **APD_30_** | 6 | 10 | 17 | 118 |
| **APD_50_** | 6 | 11 | 20 | 119 |
| **APD_90_** | 6 | 11 | 24 | 156 |
| **AMAX** | -2 | 0 | -1 | -20 |
| **RMP** | 1 | 8 | 4 | 0 |
| **Triangulation** | 8 | 13 | 33 | 193 |
| **STV** | 36 | 43 | 67 | 1161 |
| **Alternans** | 35 | 24 | 65 | 1303 |
| **ERP** | 1 | 2 | 5 | 0 |
| **Max Dispersion** | 43 | -4 | 34 | 1005 |
| **SD** | -21 | -44 | -45 | 92 |
| **Beat Escape** | 0 | 0 | 0 | 25 |
| **Refractoriness Escape** | 0 | 0 | 0 | 75 |
| **EADs** | 0 | 0 | 0 | 0 |

# 12. RESULTS LAMOTRIGINE

Averaged raw data and averaged percent change data for vehicle control and for lamotrigine at 1 and 2 Hz are summarized for each concentration tested in the tables. Data represent the mean of n=4 trabeculae tested in the experimental series.

## **12.1 SUMMARY LAMOTRIGINE AT 1Hz**

### 12.1.1: AVERAGE RAW DATA OF ACTION POTENTIAL PARAMETERS FOR VEHICLE CONTROL AND LAMOTRIGINE AT 1Hz ON VENTRICULAR TRABECULAE AS A FUNCTION OF THE MULTIPLE OF FREE ETPC (17µM)

###

| **Parameters** | **Control** | **0.6-FOLD** | **6-FOLD** | **18-FOLD** | **59-FOLD** |
| --- | --- | --- | --- | --- | --- |
| **APD_30_** | 149 | 147 | 128 | 115 | 48 |
| **APD_50_** | 197 | 197 | 169 | 152 | 66 |
| **APD_90_** | 281 | 282 | 249 | 244 | 169 |
| **AMAX** | 107 | 101 | 106 | 97 | 39 |
| **RMP** | -86 | -83 | -87 | -90 | -74 |
| **Triangulation** | 132 | 136 | 121 | 130 | 121 |
| **STV** | 0.86 | 0.60 | 0.81 | 5.08 | 3.94 |
| **Alternans** | 1.35 | 0.88 | 1.29 | 7.23 | 6.14 |
| **ERP** | 0.43 | 0.44 | 0.41 | 0.39 | 0.38 |
| **Max Dispersion** | 4.67 | 4.42 | 5.13 | 10.63 | 28.20 |
| **SD (APD_90_)** | 33 | 32 | 36 | 43 | 0 |
| **Beat Escape** | 0 | 0 | 0 | 1 | 4 |
| **Refractoriness Escape** | 0 | 0 | 0 | 0 | 0 |
| **EADs** | 0 | 0 | 0 | 0 | 0 |

### 12.1.2: AVERAGE PERCENTAGE CHANGES COMPARED TO VEHICLE CONTROL FOR ACTION POTENTIAL PARAMETERS FOR LAMOTRIGINE AT 1Hz ON VENTRICULAR TRABECULAE AS A FUNCTION OF THE MULTIPLE OF FREE ETPC (17µM)

| **Parameters** | **0.6-FOLD** | **6-FOLD** | **18-FOLD** | **59-FOLD** |
| --- | --- | --- | --- | --- |
| **APD_30_** | -2 | -15 | -23 | -69 |
| **APD_50_** | 0 | -14 | -23 | -67 |
| **APD_90_** | 1 | -11 | -13 | -38 |
| **AMAX** | -5 | -1 | -9 | -63 |
| **RMP** | -3 | 2 | 5 | -9 |
| **Triangulation** | 3 | -8 | -2 | 1 |
| **STV** | -26 | 302 | 477 | 2736 |
| **Alternans** | -35 | -5 | 435 | 354 |
| **ERP** | 4 | -3 | -7 | -10 |
| **Max Dispersion** | -5 | 10 | 127 | 503 |
| **SD** | -3 | 8 | 29 | 0 |
| **Beat Escape** | 0 | 0 | 25 | 100 |
| **Refractoriness Escape** | 0 | 0 | 0 | 0 |
| **EADs** | 0 | 0 | 0 | 0 |

###

## **12.2 SUMMARY LAMOTRIGINE AT 2Hz**

### 12.2.1: AVERAGE RAW DATA OF ACTION POTENTIAL PARAMETERS FOR VEHICLE CONTROL AND LAMOTRIGINE AT 2Hz ON VENTRICULAR TRABECULAE AS A FUNCTION OF THE MULTIPLE OF FREE ETPC (17µM)

| **Parameters** | **Control** | **0.6-FOLD** | **6-FOLD** | **18-FOLD** | **59-FOLD** |
| --- | --- | --- | --- | --- | --- |
| **APD_30_** | 105 | 107 | 103 | 110 | 106 |
| **APD_50_** | 144 | 149 | 139 | 139 | 145 |
| **APD_90_** | 228 | 232 | 221 | 227 | 268 |
| **AMAX** | 105 | 97 | 103 | 85 | 48 |
| **RMP** | -84 | -82 | -87 | -90 | -74 |
| **Triangulation** | 123 | 124 | 117 | 117 | 162 |
| **STV** | 1.59 | 0.58 | 2.93 | 18.59 | 2.81 |
| **Alternans** | 2.29 | 0.95 | 4.34 | 34.70 | 3.93 |
| **ERP** | 0.44 | 0.46 | 0.41 | 0.33 | 0.41 |
| **Max Dispersion** | 6.50 | 6.98 | 10.50 | 42.23 | 21.90 |
| **SD (APD_90_)** | 30 | 24 | 30 | 38 | 0 |
| **Beat Escape** | 0 | 0 | 1 | 4 | 4 |
| **Refractoriness Escape** | 0 | 0 | 0 | 0 | 0 |
| **EADs** | 0 | 0 | 0 | 0 | 0 |

### 12.2.2: AVERAGE PERCENTAGE CHANGES COMPARED TO VEHICLE CONTROL FOR ACTION POTENTIAL PARAMETERS FOR LAMOTRIGINE AT 2Hz ON VENTRICULAR TRABECULAE AS A FUNCTION OF THE MULTIPLE OF FREE ETPC (17µM)

| **Parameters** | **0.6-FOLD** | **6-FOLD** | **18-FOLD** | **59-FOLD** |
| --- | --- | --- | --- | --- |
| **APD_30_** | 2 | -1 | 4 | 4 |
| **APD_50_** | 3 | -3 | -4 | 7 |
| **APD_90_** | 2 | -3 | 2 | 34 |
| **AMAX** | -7 | -2 | -16 | -52 |
| **RMP** | -3 | 3 | 10 | -6 |
| **Triangulation** | 2 | -4 | -1 | 64 |
| **STV** | -6 | 262 | 1720 | 870 |
| **Alternans** | -59 | 90 | 1417 | 72 |
| **ERP** | 4 | -7 | -25 | -7 |
| **Max Dispersion** | 7 | 62 | 550 | 237 |
| **SD** | -22 | 0 | 27 | 0 |
| **Beat Escape** | 0 | 25 | 100 | 100 |
| **Refractoriness Escape** | 0 | 0 | 0 | 0 |
| **EADs** | 0 | 0 | 0 | 0 |

# 13. RESULTS MEXILETINE

Averaged raw data and averaged percent change data for vehicle control and for mexiletine at 1 and 2 Hz are summarized for each concentration tested in the tables. Data represent the mean of n=4 trabeculae tested in the experimental series.

## **13.1 SUMMARY MEXILETINE AT 1Hz**

### 13.1.1: AVERAGE RAW DATA OF ACTION POTENTIAL PARAMETERS FOR VEHICLE CONTROL AND MEXILETINE AT 1Hz ON VENTRICULAR TRABECULAE AS A FUNCTION OF THE MULTIPLE OF FREE ETPC (4.1µM)

###

| **Parameters** | **Control** | **0.7-FOLD** | **7-FOLD** | **24-FOLD** | **73-FOLD** |
| --- | --- | --- | --- | --- | --- |
| **APD_30_** | 183 | 174 | 158 | 137 | 120 |
| **APD_50_** | 239 | 231 | 206 | 180 | 165 |
| **APD_90_** | 324 | 321 | 297 | 282 | 277 |
| **AMAX** | 112 | 111 | 109 | 102 | 86 |
| **RMP** | -90 | -91 | -91 | -89 | -76 |
| **Triangulation** | 141 | 147 | 140 | 145 | 157 |
| **STV** | 0.53 | 0.51 | 0.49 | 12.96 | 1.74 |
| **Alternans** | 0.68 | 0.72 | 0.68 | 17.51 | 2.53 |
| **ERP** | 0.43 | 0.43 | 0.41 | 0.39 | 0.42 |
| **Max Dispersion** | 3.75 | 3.75 | 2.82 | 50.33 | 6.30 |
| **SD (APD_90_)** | 24 | 17 | 15 | 7 | 19 |
| **Beat Escape** | 0 | 1 | 1 | 3 | 1 |
| **Refractoriness Escape** | 0 | 0 | 0 | 0 | 0 |
| **EADs** | 0 | 0 | 0 | 0 | 0 |

### 13.1.2: AVERAGE PERCENTAGE CHANGES COMPARED TO VEHICLE CONTROL FOR ACTION POTENTIAL PARAMETERS FOR MEXILETINE AT 1Hz ON VENTRICULAR TRABECULAE AS A FUNCTION OF THE MULTIPLE OF FREE ETPC (4.1µM)

| **Parameters** | **0.7-FOLD** | **7-FOLD** | **24-FOLD** | **73-FOLD** |
| --- | --- | --- | --- | --- |
| **APD_30_** | -5 | -13 | -25 | -27 |
| **APD_50_** | -3 | -14 | -25 | -27 |
| **APD_90_** | -1 | -8 | -13 | -9 |
| **AMAX** | 0 | -3 | -9 | -22 |
| **RMP** | 1 | 1 | -1 | -13 |
| **Triangulation** | 4 | -1 | 3 | 12 |
| **STV** | 1 | -3 | 2873 | 182 |
| **Alternans** | 7 | 0 | 2481 | 272 |
| **ERP** | 1 | -5 | -8 | -1 |
| **Max Dispersion** | 0 | -25 | 1242 | 68 |
| **SD** | -28 | -37 | -70 | -20 |
| **Beat Escape** | 25 | 25 | 75 | 50 |
| **Refractoriness Escape** | 0 | 0 | 0 | 0 |
| **EADs** | 0 | 0 | 0 | 0 |

###

## **13.2 SUMMARY MEXILETINE AT 2Hz**

### 13.2.1: AVERAGE RAW DATA OF ACTION POTENTIAL PARAMETERS FOR VEHICLE CONTROL AND MEXILETINE AT 2Hz ON VENTRICULAR TRABECULAE AS A FUNCTION OF THE MULTIPLE OF FREE ETPC (4.1µM)

| **Parameters** | **Control** | **0.7-FOLD** | **7-FOLD** | **24-FOLD** | **73-FOLD** |
| --- | --- | --- | --- | --- | --- |
| **APD_30_** | 137 | 126 | 128 | 129 | 82 |
| **APD_50_** | 181 | 172 | 167 | 169 | 118 |
| **APD_90_** | 262 | 257 | 255 | 269 | 234 |
| **AMAX** | 108 | 105 | 99 | 90 | 43 |
| **RMP** | -88 | -88 | -89 | -88 | -68 |
| **Triangulation** | 125 | 131 | 127 | 139 | 152 |
| **STV** | 0.91 | 1.38 | 0.76 | 21.80 | 15.46 |
| **Alternans** | 1.26 | 2.02 | 1.03 | 30.91 | 21.46 |
| **ERP** | 0.43 | 0.45 | 0.39 | 0.37 | 0.45 |
| **Max Dispersion** | 7.12 | 6.13 | 4.70 | 59.90 | 35.75 |
| **SD (APD_90_)** | 30 | 13 | 24 | 22 | 93 |
| **Beat Escape** | 0 | 2 | 3 | 4 | 2 |
| **Refractoriness Escape** | 0 | 0 | 0 | 0 | 0 |
| **EADs** | 0 | 0 | 0 | 0 | 0 |

### 13.2.2: AVERAGE PERCENTAGE CHANGES COMPARED TO VEHICLE CONTROL FOR ACTION POTENTIAL PARAMETERS FOR MEXILETINE AT 2Hz ON VENTRICULAR TRABECULAE AS A FUNCTION OF THE MULTIPLE OF FREE ETPC (4.1µM)

| **Parameters** | **0.7-FOLD** | **7-FOLD** | **24-FOLD** | **73-FOLD** |
| --- | --- | --- | --- | --- |
| **APD_30_** | -7 | -6 | -4 | -30 |
| **APD_50_** | -4 | -8 | -7 | -30 |
| **APD_90_** | -1 | -2 | 3 | -3 |
| **AMAX** | -2 | -8 | -16 | -60 |
| **RMP** | 0 | 1 | 0 | -19 |
| **Triangulation** | 5 | 2 | 11 | 25 |
| **STV** | 81 | 3 | 2766 | 3767 |
| **Alternans** | 59 | -19 | 2347 | 1598 |
| **ERP** | 5 | -9 | -13 | 5 |
| **Max Dispersion** | -14 | -34 | 741 | 402 |
| **SD** | -58 | -20 | -29 | 210 |
| **Beat Escape** | 50 | 75 | 100 | 100 |
| **Refractoriness Escape** | 0 | 0 | 0 | 0 |
| **EADs** | 0 | 0 | 0 | 0 |
